# A Computational Workflow for Probabilistic Quantitative *in Vitro* to *in Vivo* Extrapolation

**DOI:** 10.3389/fphar.2018.00508

**Published:** 2018-05-18

**Authors:** Kevin McNally, Alex Hogg, George Loizou

**Affiliations:** Health and Safety Executive, Buxton, United Kingdom

**Keywords:** *in vitro*, *in vivo*, extrapolation, PBPK, benchmark dose, computational, workflow

## Abstract

A computational workflow was developed to facilitate the process of quantitative *in vitro* to *in vivo* extrapolation (QIVIVE), specifically the translation of *in vitro* concentration-response to *in vivo* dose-response relationships and subsequent derivation of a benchmark dose value (BMD). The workflow integrates physiologically based pharmacokinetic (PBPK) modeling; global sensitivity analysis (GSA), Approximate Bayesian Computation (ABC) and Markov Chain Monte Carlo (MCMC) simulation. For a given set of *in vitro* concentration and response data the algorithm returns the posterior distribution of the corresponding *in vivo*, population-based dose-response values, for a given route of exposure. The novel aspect of the workflow is a rigorous statistical framework for accommodating uncertainty in both the parameters of the PBPK model (both parameter uncertainty and population variability) and in the structure of the PBPK model itself recognizing that the model is an approximation to reality. Both these sources of uncertainty propagate through the workflow and are quantified within the posterior distribution of *in vivo* dose for a fixed representative *in vitro* concentration. To demonstrate this process and for comparative purposes a similar exercise to previously published work describing the kinetics of ethylene glycol monoethyl ether (EGME) and its embryotoxic metabolite methoxyacetic acid (MAA) in rats was undertaken. The computational algorithm can be used to extrapolate from *in vitro* data to any organism, including human. Ultimately, this process will be incorporated into a user-friendly, freely available modeling platform, currently under development, that will simplify the process of QIVIVE.

## Introduction

The prospect of an animal-free, *in vitro* bioassay based, human safety testing of chemicals strategy was increased with the publication of the US National Research Council (NRC) report titled “Toxicity Testing in the 21st Century: A Vision and a Strategy” (NRC, 2007). Considerable impetus for this vision occurred following enforcement of the EU Cosmetics Regulation (EC 1223/2009) in 2013 which imposed a full marketing ban in Europe for cosmetic products and ingredients tested on animals anywhere in the world (Coecke et al., 2013). However, to date the development of a reliable non-animal, *in vitro* bioassay based testing strategy for human safety testing of chemicals is still regarded as the holy grail (Louisse et al., 2016). Aside from the issue of better concordance between *in vitro* and *in vivo* human toxicity endpoints a number of fundamental problems with *in vitro* cell systems remain to be resolved, such as the artificial conditions in which they are maintained (see Hartung and Daston, 2009 for more details). Nevertheless, encouraging developments in *in vitro* systems and efforts to exploit the information generated using them continue to be reported (Shintu et al., 2012; Alépée et al., 2014; Bahinski, 2015; Hartung, 2017; Ramirez et al., 2017; Schmidt et al., 2017).

The determination of a reference point, also known as a point of departure (PoD), such as the benchmark dose (BMD) and no-observed-adverse-effect-level (NOAEL) from *in vitro* concentration-response data is a pre-requisite for regulatory use. Indeed, a characteristic often ascribed to technologies that are quickly adopted is that they are compatible with existing practices (Jönsson, 2016). Therefore, *in vitro* concentration-response data must be converted to *in vivo* dose-responses from which a PoD may be derived in order to have any utility in human safety testing of chemicals. The term quantitative *in vitro* to *in vivo* extrapolation (QIVIVE) is used to describe efforts addressing this problem (Bale et al., 2014; Hartung, 2017) and the use of PBPK^1^ modeling-based reverse dosimetry for the translation of *in vitro* to *in vivo* responses represents a significant part of the solution (Louisse et al., 2012, 2016; Bessems and Geraets, 2013; Coecke et al., 2013; Strikwold et al., 2013, 2017a,b; Bessems et al., 2014; McNally and Loizou, 2015; Boonpawa et al., 2017; Li et al., 2017; Punt et al., 2017).

There have been a number of studies in which PBPK modeling-based reverse dosimetry and statistical techniques were used to reconstruct *in vivo* exposure or dose consistent with, (1) human biological monitoring (BM) data, and (2) *in vitro* concentration-response curves. In the case of BM studies *in vivo* exposure or dose was reconstructed at both the individual and population level (Georgopoulos et al., 1994; Roy and Georgopoulos, 1998; Tan et al., 2006a,b; Liao et al., 2007; Clewell et al., 2008; Lyons et al., 2008; Mosquin et al., 2009; McNally et al., 2012). Population-based estimates of exposure that account for human inter-individual variability, both in the modeling of chemical disposition in the body and in the description of plausible exposure conditions, was achieved using Bayesian inference (Lyons et al., 2008). Gelman et al. (1996) used a Bayesian approach as a general method of parameter estimation in PBPK models. This method was originally used for model calibration (Bernillon and Bois, 2000; Jonsson and Johanson, 2001; Hack, 2006; Covington et al., 2007). Lyons et al. (2008) extended PBPK model calibration to include the unique exposure for each individual as another parameter to be estimated, alongside two additional “hyper-parameters,” the mean and standard deviation of exposures at the population level, to model variability in exposure. In this way the model could be applied to interpret population-based BM data. The linking of a PBPK model with Bayesian inference has a number of advantages with regard to exposure or dose reconstruction. Firstly, it is an appropriate approach for systems where tissue dose is not necessarily linearly related to external exposure. Secondly, defining informative prior distributions around parameters converts a deterministic model to a population model, thereby accounting for inter-individual variability. Thirdly, this combination can extract population variability and multiple routes of exposure information integrated within pharmacokinetic data (McNally et al., 2012).

On the other hand the majority of studies reporting the translation of *in vitro* concentration-response to *in vivo* dose-response curves used a different approach more accurately described as “iterative forward dosimetry.” This approach assumes the model is an accurate emulation of reality in which all parameters, other than input dose or exposure, are fixed. The latter only are altered within an optimization routine to estimate a target *in vivo* concentration which has the same magnitude as the measured *in vitro* concentration. The dose concentration which corresponds to the target *in vitro* concentration is considered to be a surrogate for the *in vivo* concentration (Louisse et al., 2012, 2016; Strikwold et al., 2013, 2017a,b; Wambaugh et al., 2015; Boonpawa et al., 2017; Li et al., 2017). The interpretation of reconstructed doses and exposures derived in this way can be problematic. Firstly, the results of any sensitivity analysis of the model were not used in the *in vitro* to *in vivo* conversion process. In most models there will be several sensitive parameters that have a significant impact on model output. This means small changes in the magnitude of any sensitive parameter may have a significant impact on output, in this case, reconstructed dose or exposure concentration. It is therefore possible that the reconstructed dose or exposure estimated with and without incorporation of sensitive parameters can be quite different. A second issue is with the presumed accuracy of the PBPK model. An external dose that provides a desired *in-vitro* concentration—typical dose metrics are peak concentration or area under the curve (AUC) for parent chemical or some metabolite in venous blood (as a surrogate for the *in-vitro* concentration)—is computed. Only a very small error, within the user-specified tolerance limits of the optimization routine is associated with this QIVIVE translation. The use of a specific value from the simulation for direct use in risk assessment, in effect assumes a higher degree of accuracy of the PBPK model than is required in the more traditional use of such models. The model is (implicitly) interpreted as an adequate surrogate for the human or animal rather than as a useful modeling tool. Crucially, there is no framework for addressing model inadequacy. This is important since the level of detail (fidelity) captured in the model could have a bearing on model output (Rowland et al., 2017). Thirdly, the iterative forward dosimetry approach uses a deterministic model with one set of model parameters only. This is equivalent to using a single individual as a representative of all people in the safety testing of chemicals.

The importance in understanding and quantifying the level of uncertainty in each step of a chemical safety assessment with non-animal methods has recently been emphasized (Berggren et al., 2017).

In this report we describe the adaptation of a workflow developed previously for the reconstruction of exposure from BM data (McNally et al., 2012). We used PBPK modeling, Approximate Bayesian Computation (ABC) and Markov Chain Monte Carlo (MCMC) simulation to convert *in vitro* concentration-response data to *in vivo* dose-response data. To demonstrate this process we undertook a similar exercise to Louisse et al. (2010) although with the added objective of accommodating model and parameter value uncertainty within an efficient modeling framework.

The motivation for this work is twofold: (1) development of a rigorous statistical framework for accommodating uncertainty in both the parameters of the PBPK model and lack of fit of the model to measured data, and a consideration of how this affects an *in-vivo* dose response relationship, and (2) to develop a workflow and code that will be incorporated into a user-friendly, freely available modeling platform called RVis, currently under development, that will simplify the process of translation of *in vitro* concentration-response to *in vivo* dose-response relationships^2^.

## Methods

### PBPK model

#### Software

The generic PBPK model code describing the kinetics of glycol ethers (Louisse et al., 2010) was provided by Dr. Jochem Louisse^3^ in CSL, the equation-based language implemented in acslX^TM^ software. However, support for acslX^TM^ was discontinued in November 2015 (Lin et al., 2017). Therefore, the CSL code was translated into the R language (R Development Core Team, 2008) using ACSL2R (http://acsl2r.hslmathsci.org/) and run using RStudio (R Studio Team, 2016).

In order to perform GSA the model code was further modified to ensure that logical constraints on mass balance and blood flow to the tissues were met by adopting the re-parameterizations described in Gelman et al. (1996).

PBPK models were solved using the deSolve package of R. GSA of model outputs [Morris screening test and extended Fourier Amplitude Sensitivity Test (eFAST)] were conducted using the Sensitivity package of R. Reshaping of data and plotting using the reshape and ggplot2 packages respectively. The md2c package was used to induce rank correlations in samples (Wickham, 2007; Pouillot and Delignette-Muller, 2010; Soetaert et al., 2010; Pujol and Iooss, 2015). The main effects and total effects (McNally et al., 2011) were computed at each time point and parameter sensitivities were studied over this period using Lowry plots generated as described in McNally et al. (2011).

Benchmark dose values (BMDs) were calculated using PROAST version 65.0 (proast@rivm.nl) and R version 3.4.1 (https://cran.r-project.org/bin/windows/base/old/3.4.1/).

All plots were created using R and gglot2 (R Development Core Team, 2008; Wickham, 2009).

#### Data

Measured plasma concentrations of methoxyacetic acid (MAA), a metabolite of ethylene glycol monoethyl ether (EGME), in exposed rats were obtained by digitizing **Figure 4** in Hays et al. (2000) and **Figure 2** in Gargas et al. (2000). These data were used by Louisse et al. (2010) and in this study to evaluate the PBPK model. These data were digitized using DigitizeIt Version 2.0.6 (www.digitizeit.de).

The *in vitro* embryotoxic effect data determined in a study by de Jong et al. (2009) as described in Louisse et al. (2010) were also kindly supplied by Dr. Jochem Louisse. The *in vitro* concentrations of MAA were, 0, 0.3, 0.6, 1.1, 2.8, 5.5, 11.1 (mM) conducted in triplicate in two different laboratories, therefore, six tests in total.

Following conversion to *in vivo* dose responses the data were used to calculate BMDs.

#### Hardware

The computer used in this study was a Dell Optiplex 9020 with an Intel(R) Core ™ i5-4590 CPU @ 3.30 GHz with 8.00 GB RAM running Windows 7 Enterprise Service Pack 1.

### Description of approach

**Figure 1:**
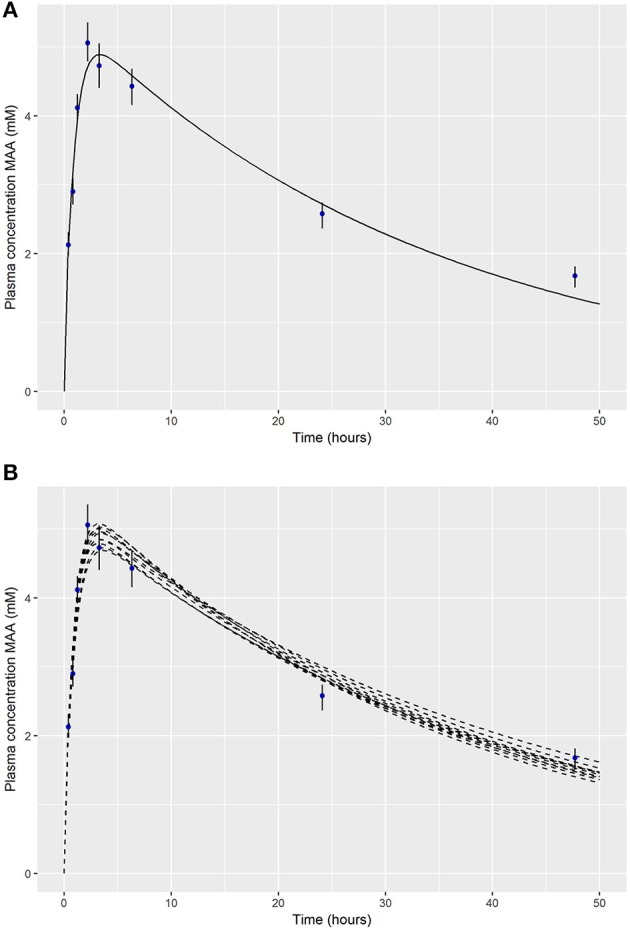
**(A)** Comparison of PBPK model predictions of MAA in venous blood against the experimental data of Hays et al. (2000) (upper panel); **(B)** a comparison of 10 alternative parameter sets against the experimental data of Hays et al. (2000) following an oral dose of 3.3 mmol/kg bodyweight EGME (lower panel).

The original work described herein focusses on one specific model described in Louisse et al. (2010): the rat model following a single dose oral exposure and a repeat (5 day) inhalation exposure to EGME. As a motivation for the approach set out in detail in Appendix 1 we re-evaluate the performance of the EGME PBPK model following an oral dose of 3.3 mmol/kg bodyweight. Figure 1A, adapted from Figure 2B of Louisse et al. (2010) shows a comparison of the PBPK model estimates (populated with baseline PBPK model parameters) against the experimental data of Hays et al. (2000).

**Figure 2:**
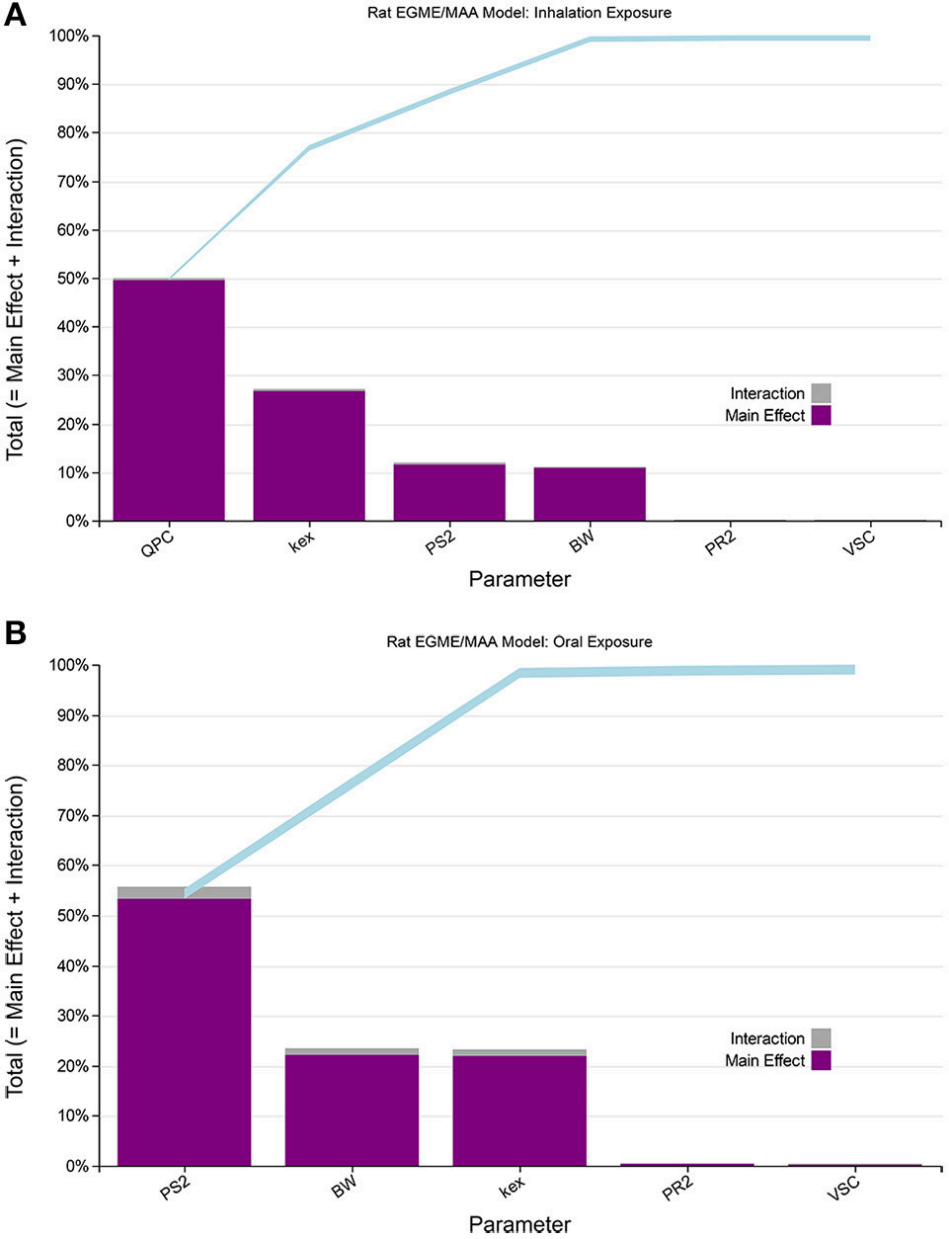
Lowry plots of the eFAST quantitative measure of the most sensitive parameters identified by Morris screening. The total effect of a parameter S_Ti_ comprised the main effect S_i_ (purple bar) and any interactions with other parameters (gray bar) given as a proportion of variance. The ribbon, representing variance due to parameter interactions, is bounded by the cumulative sum of main effects (lower bold line) and the minimum of the cumulative sum of the total effects (upper bold line) **(A)** for venous blood MAA concentrations following, **(A)** inhalation exposure (upper panel), and **(B)** oral exposure (lower panel).

Whilst the PBPK model is a good fit to the experimental data (Figure 1A) the model underestimates the maximum concentration observed in experimental data., Alternative parameter combinations, corresponding to modest changes to the assumed physiology of the rat and chemical specific properties, provide a similar quality of fit to the experimental data (Figure 1B) although with a different C_max_ values^4^. Whilst only modest perturbations to the baseline assumptions of the physiology of a laboratory bred rodent might be considered reasonable, uncertainty in the physico-chemical (substance dependent) parameters of the PBPK model is more considerable. Uncertainty in the parameters of the model, *parameter value uncertainty*, should translate into uncertainty in the external oral dose corresponding to a given C_max_ (or an alternative end-point). A second source of uncertainty which it is desirable to consider is *model uncertainty*: the PBPK model is an imperfect approximation to reality therefore a model that provides an exact match to a target *in-vitro* dose is, perhaps counter-intuitively, not desirable as it leads to an underestimate of the range of external doses that are consistent with a target *in-vitro* concentration representative of an *in vivo* concentration.

The modeling framework comprises a four-step approach and is described in greater detail in the Appendix 1:

1. GSA of MAA concentrations in venous blood with conservative yet credible ranges for the model parameters to identify the key parameters for further consideration;
2. Refinement of the parameter ranges through a reverse dosimetry-type approach so that plausible limits on the varying model parameters that are consistent with the data of Hays et al. (2000) (single dose, oral exposure) and Gargas et al. (2000) (repeat dose, inhalation exposure) can be estimated.
3. Estimation of a distribution of oral dose and inhalation concentrations corresponding to each of the six experimental *in vitro* concentrations and accounting for model and parameter value uncertainty. Here we introduce a novel approach based upon Approximate Bayesian Computation.
4. Estimation of a point of departure, taken to be the benchmark dose (BMDL_10_) lower bound in the *in vivo* dose response relationship.

### Script files to implement conversion of *in vitro* concentration to *in vivo* dose response

See Appendix 2 for a description of the files used to implement this workflow.

### Calculation of *in vivo* benchmark dose

Three embyrotoxicity (inhibition of embryionic stem cell differentiation) tests were carried out in two different laboratories giving a total of six quantal datasets (de Jong et al., 2009). There were six *in vitro* concentrations of MAA in each dataset. Therefore, six *in vitro* concentrations were converted to *in vivo* doses for the inhalation and oral routes, respectively, and were used as inputs for benchmark dose analysis. To account for possible systematic differences in measured response between laboratories “laboratory” was modeled as a covariate. The *in vivo* dose-response curves were predicted using identical exposure regimes to those used to evaluate the model i.e., a single dose oral exposure to EGME and a repeat (5 day) 6 h inhalation exposure to EGME. For the uploaded data PROAST fitted 10 candidate models that were suitable for quantile response data. The benchmark dose lower bound corresponding to the most conservative model that provided an adequate fit (as assessed by the software) to the data was taken from the output. A benchmark response of 0.1 was specified.

## Results

### GSA

In the first phase of sensitivity analysis a screening analysis using the Morris test was undertaken with 25 parameters (Table 1) varied following inhalation and oral exposures to EGME. Based upon this analysis (example results are shown in Supplementary Material Tables S1, S2 and Figures S1, S2) the majority of parameters were observed to have a negligible effect on MAA in venous blood, .with six and eleven parameters for inhalation and oral dose respectively, taken forward into the second phase of sensitivity analysis using the quantitative eFAST technique.

**Table 1:** Physiological parameters used in the PBPK model for ethylene glycol monomethyl ether adapted from Gargas et al. (2000) and Louisse et al. (2010).

**Physiological parameters**	**Abbreviation**	**Mean**	**15%**	**Minimum**	**Maximum**
Body weight (kg)	BW	0.237^a^	0.036	0.201	0.273
**% BW**					
Liver	VLC	4	0.600	3.400	4.600
Fat	VFC	10.1	1.515	8.585	11.615
Slowly perfused tissue	VSC	65	9.750	55.250	74.750
Rapidly perfused tissue	VRC	6.1	0.915	5.185	7.015
Blood	VBC	5.9	0.885	5.015	6.785
Cardiac output (L h^−1^ kg^−1^ BW)	QCC	14	2.100	11.900	16.100
Alveolar ventilation (L h^−1^ kg^−1^ BW)	QPC	14	2.100	11.900	16.100
**% CARDIAC OUTPUT**					
Liver	QLC	25	3.750	21.250	28.750
Fat	QFC	14.2	2.130	12.070	16.330
Slowly perfused tissue	QSC	15	2.250	12.750	17.250
Rapidly perfused tissue	QRC	45.8	6.870	38.930	52.670
**PARTITION COEFFICIENTS (EGME)**					
Blood:air	PB	32800	4920	27880	37720
Liver:blood	PL	0.76	0.114	0.646	0.874
Fat:blood	PF	0.37	0.056	0.315	0.426
Slowly perfused tissue:blood	PS	0.8	0.120	0.680	0.920
Rapidly perfused tissue:blood	PR	0.76	0.114	0.646	0.874
**PARTITION COEFFICIENTS (MAA)**					
Liver:blood	PL2	0.76	0.114	0.646	0.874
Fat:blood	PF2	0.13	0.020	0.111	0.150
Slowly perfused tissue:blood	PS2	0.8	0.120	0.680	0.920
Rapidly perfused tissue:blood	PR2	0.76	0.114	0.646	0.874
**METABOLIC RATE CONSTANTS**					
Michaelis Menten constant (mM)	K_M_	6.3	0.945	5.355	7.245
Limiting rate of metabolism (nmol h^−1^ 10^6^ hepatocytes)	V_Max_	1511	226.65	1284.35	1737.65
Urinary excretion rate (L h^−1^)	K_ex_	0.0045	0.001	0.004	0.005
Oral uptake rate (h^−1^)	K_up_	4	0.600	3.400	4.600

a *Estimated as a function of time (hours) where T > 312 h as described in Gargas et al. (2000)*.

Four parameters, QPC (alveolar ventilation rate), K_ex_ (urinary excretion rate), PS2 (MAA blood:air partition coefficient) and BW (body weight), accounted for almost 100% of variance in venous blood MAA during inhalation exposure to EGME during the entire simulation of 200 h (Supplementary Material, Figure S3). The ranking of the parameters, on the basis of proportional contribution to variance, changed throughout the simulation. Initially, PS2 was dominant for the first 20 h to be replaced by QPC. Figure 2A, shows the rankings at 105 h, that is, 3 h post-exposure. Following single dose oral exposure the eFAST results (Supplementary Material, Figure S4) indicated variance was initially dominated by parameters governing the rate of metabolism of parent chemical (EGME), particularly Vmax. However, after the first hour of exposure parameter PS2 was dominant. The influence of BW and K_ex_, increased over time with three parameters PS2, BW and K_ex_ accounting for almost 100% of variance in venous blood MAA during oral exposure to EGME during the period of 4 up to 50 h following exposure (covering the period where peak concentration of MAA was reached and the subsequent elimination phase. PS2 was initially dominant for the first 19 h before switching in ranking started to occur. Figure 2B, shows the rankings at 19 h post oral administration.

Therefore, four parameters, QPC, K_ex_, PS2, and BW, for inhalation and three, PS2, BW, and K_ex_, for oral exposure were used in the ABC-MCMC simulations to convert *in vitro* concentrations to *in vivo* doses.

### Refinement

Modification of model parameters was achieved through a Bayesian calibration approach as described in the section on Refinement (Appendix 1). The posterior median and a 95% credible interval for the four and three retained parameters for the inhalation and oral exposures to EGME are given in Table 2.

**Table 2:** Posterior medians and 95% confidence intervals for calibrated parameters.

**Parameter**	**Oral dose refinement**		**Inhalation dose refinement**	
	**Median**	**95% interval**	**Median**	**95% interval**
BW	0.250	0.245–0.255	0.249	0.245–0.254
QPC	NA		12.46	11.92–15.27
PS2	0.746	0.662–0.835	0.724	0.527–0.900
kex	0.0041	0.0038–0.0047	0.0049	0.0042–0.0052

#### Inhalation dose

**Figure 3:**
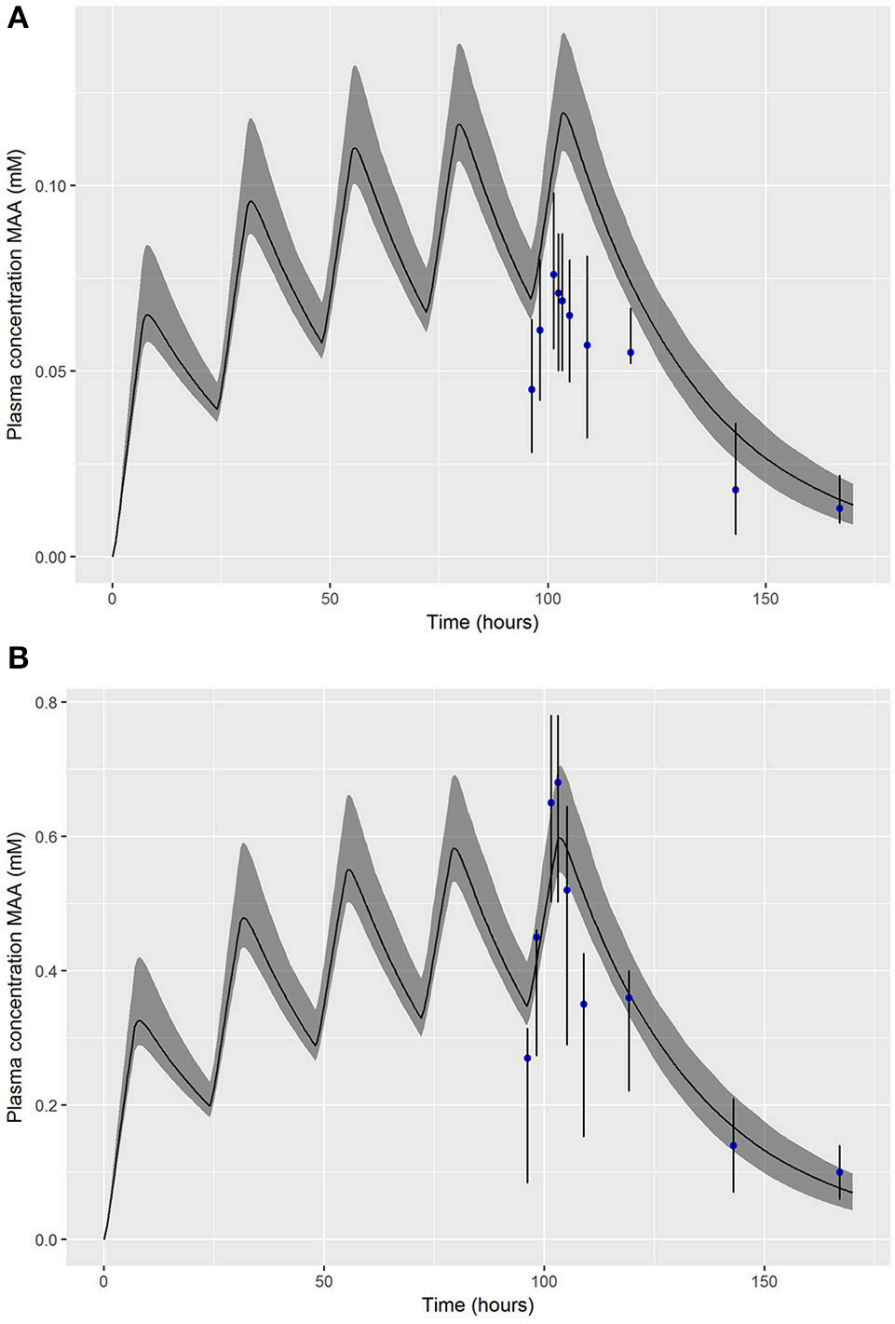
Posterior mode and a 95% credible interval for the exposure-time concentration of MAA following a 5 day inhalation exposure to 10 ppm **(A)** and 50 pmm **(B)** EGME.

The PBPK model was run (for a simulation period of 170 h) for each of the retained parameter sets and for concentrations of both 10 and 50 ppm. At each time point the predictions of the plasma concentration of MAA were ordered and the 2.5, 50, and 97.5th of the ordered values were read off—the dataset of 50th percentiles at each time point is a point estimate (posterior median) of the plasma concentration of MAA over the simulation period whereas the 2.5 and 97.5th percentiles correspond to an approximate 95% prediction interval for the plasma concentration of MAA over the simulation period. Figure 3 shows a comparison of these summary statistics with the experimental (calibration) data of Gargas et al. (2000). The fit to data at 10 ppm was poor; the models did not capture the trend of the observations (Figure 3A). The experimental data at 50 ppm were not fully enveloped by the 95% credible interval calculated from MCMC output which reflects the large variability across the replicate animals at each time point in the experimental data and the sensitivity to outliers.However, the overall fit of the spread of models (indexed by the different parameter sets) was consistent with the assumed log-normal error distribution (Figure 3B).

In order to test the ability of a different statistical to measure model structure uncertainty a variant case with a Gaussian error model was considered for the calibration to inhalation data at 10 and 50 ppm. A slightly improved fit to the experimental data at 10 ppm was achieved with this model however the inconsistency between the PBPK model and experimental data was not resolved (Supplementary Material, Figure S5). This result is consistent with the fit shown to these data in Louisse et al. (2010); the data would indicate a more rapid clearance of MAA at low dose for a repeat dose inhalation study than can be achieved through variation of model parameters alone, and suggests a structural problem in the model for lower doses. The predictions at 50 ppm were thought more relevant for the target concentrations in this work therefore calibration was repeated using just model and data for the 50 ppm experiment. The results in Table 2 reflect a calibration to the 50 ppm data using the log-normal error model (1) (see Appendix 1).

Wide ranges (of ±15% the median, Table 1) were assumed for PS2 and k_ex_. Through the process of calibration and the constraint imposed by the relatively tight range on BW, the ranges for these two parameters were substantially reduced (Table 2).

#### Oral dose

**Figure 4:**
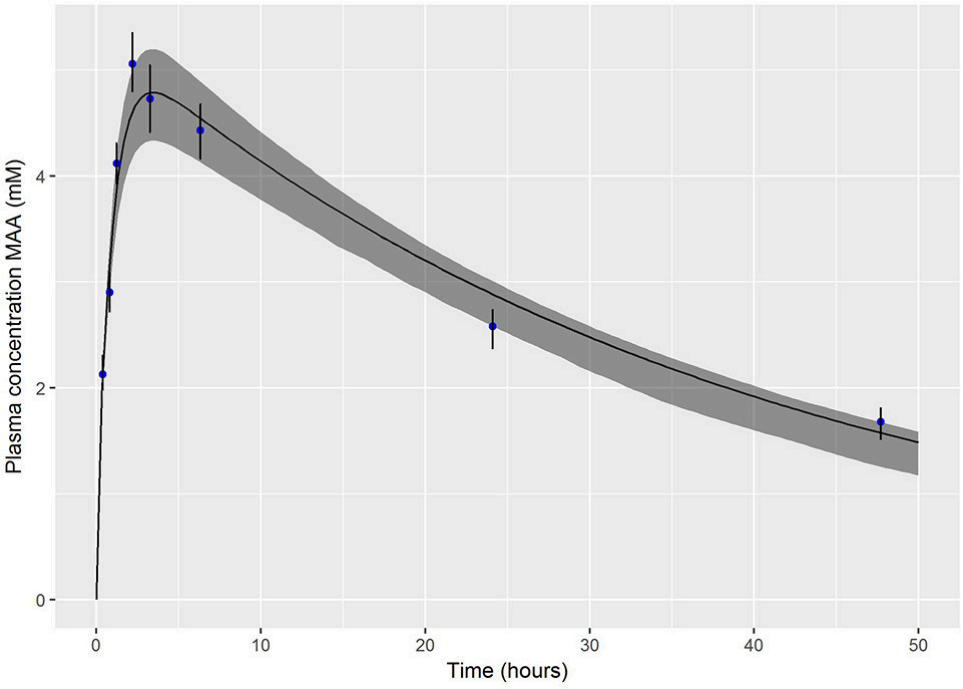
Posterior mode and a 95% credible interval for the exposure-time concentration of MAA following and oral dose of 3.3 mmol/kg bodyweight EGME.

The PBPK model was run (for a simulation period of 50 h) for each of the retained parameter sets. The median and a 95% prediction interval were calculated as described above. Figure 4 shows a comparison of these summary statistics with the experimental (calibration) data of Hays et al. (2000). The experimental data are enveloped by the 95% credible interval calculated from MCMC output.

The summary statistics for PS2 and k_ex_ (Table 2) indicated that calibration substantially reduced uncertainty in these parameters compared with the assumed (vague) prior distributions.

### ABC

**Figure 5:**
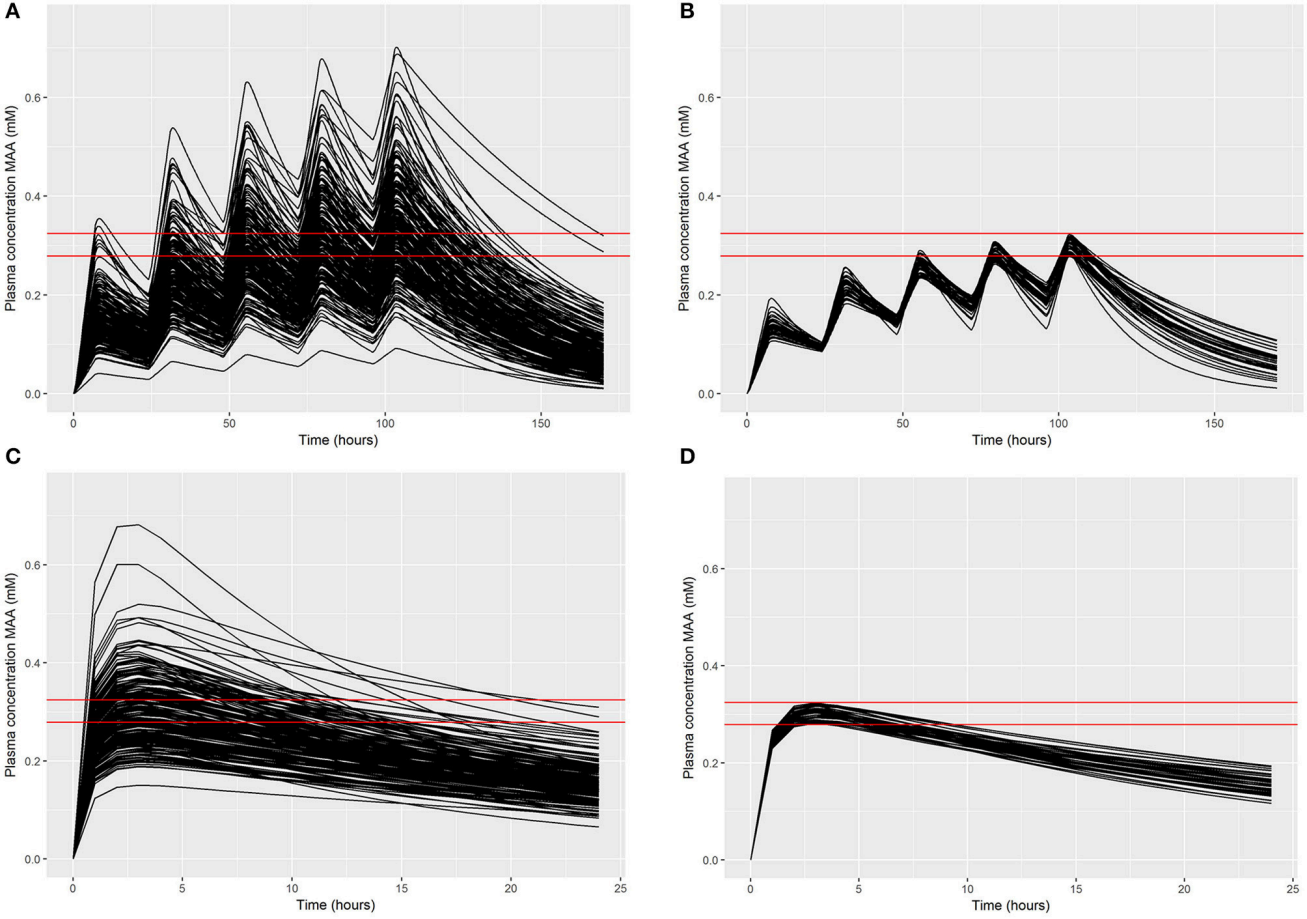
Comparisons of the 200 concentration response profiles simulated in the rejection phase for a target concentration of 0.28 mM: **(A)** 200 exposure-time concentrations of MAA following a 5 day inhalation exposure (upper left panel); **(B)** retained samples within a relative error of 7.5% (upper right panel); **(C)** 200 exposure-time concentrations of MAA following an oral dose (lower left panel); **(D)** retained samples within a relative error of 7.5% (lower right panel).

In the first phase of the ABC approach 200 simulations were run for each dose concentration. A comparison of the exposure time-concentrations for the 200 simulations for the lowest target dose concentration is made in Figures 5A,C for inhalation and oral dose respectively, and demonstrates the wide range of behaviors—this pattern is broadly representative of all dose concentrations. In the figures the solid red lines represent the relative error of 7.5% of C_max_. Figures 5B,D show just the simulations that satisfied the acceptance criteria for inhalation and oral dose respectively, and were retained for subsequent inference. Figures 5B,D show the retained simulations converge at the peak (noting the peak refers to the fifth day of exposure for the inhalation simulations) however the subsequent rate of elimination of MAA varied widely over the retained simulations. A comparison of simulated and retained exposure time-concentrations for the simulations at all six dose concentrations is made in Supplementary Material Figures S6–S8 (repeat dose inhalation) and Figures S9–S11 (single dose oral).

Approximately 20% of the simulations were within a relative error of 7.5% for each target plasma concentration following inhalation exposure, whereas approximately 25% of simulations were retained following oral exposure; this difference likely results from the lower number of sensitive parameters for the oral exposure model. In all cases there was a reasonable retained sample for estimating a covariance matrix for use in the subsequent ABC MCMC.

Due to the approach for sampling initial values, the MCMC algorithm was initialized within the posterior distribution (for each route of exposure and target plasma concentration) therefore a burn-in was unnecessary. Two chains, with different initial values, were run in parallel on different cores (making use of the parallel environment within R) for each dose concentration. The acceptance rate was approximately 60% for each chain with only modest autocorrelations between subsequent iterations, demonstrating relatively weak dependence on the current state of the chain. Every second sample was retained for subsequent analysis. The 2,500 retained samples resulting from each of the two chains were pooled for each target dose.

**Figure 6:**
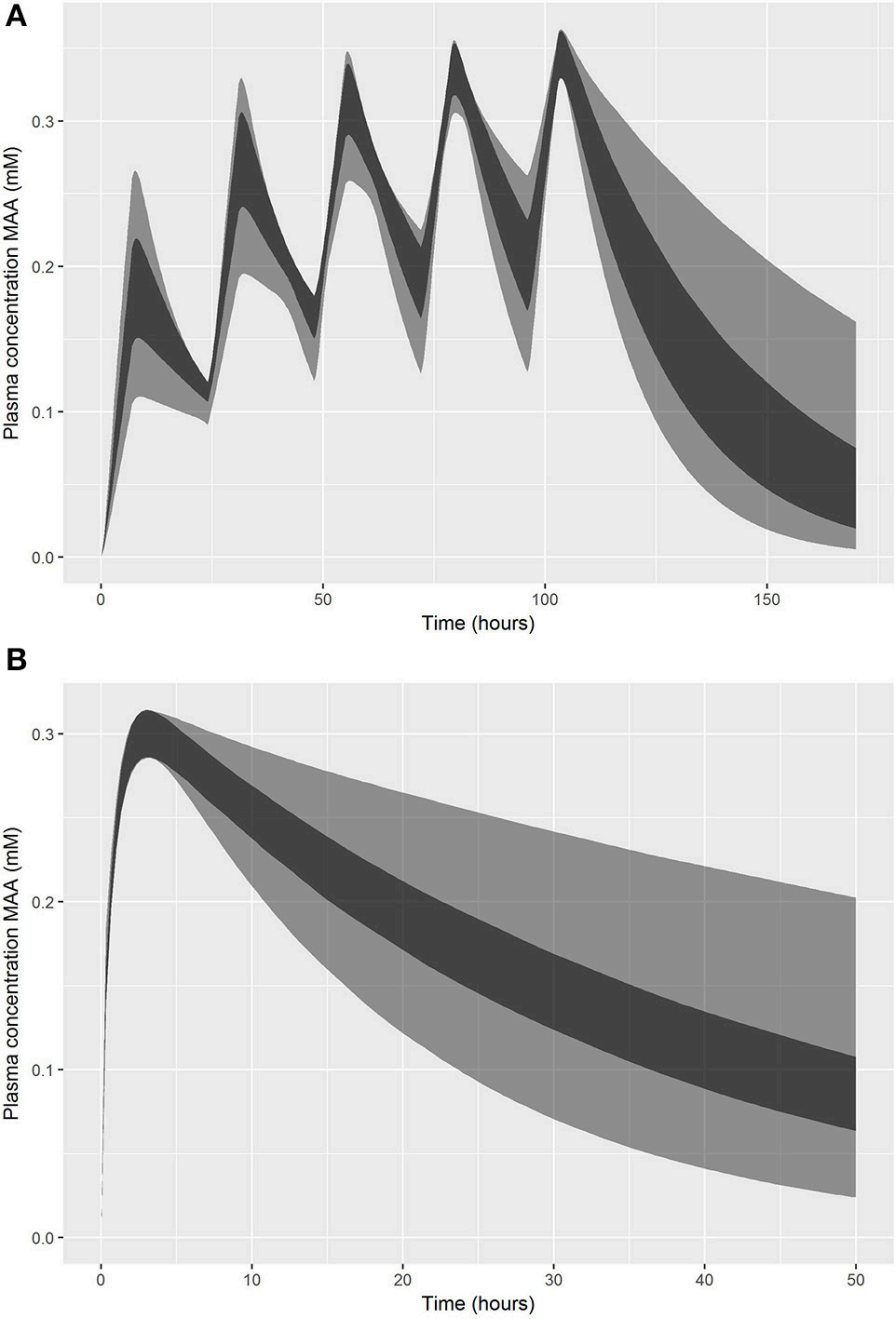
Comparison of 95% credible intervals for concentration-responses within a relative error of 5% (lighter interval) and based on only those samples with parameters within the calibrated limits (darker interval): **(A)** exposure-time concentrations of MAA following a 5 day inhalation exposure (upper panel); **(B)** exposure-time concentrations of MAA following an oral dose (lower panel).

Summary statistics were based on the retained 5,000 simulations and are given in Table 3. A comparison of the 95% credible intervals for QPC, K_ex_ and PS2 following inhalation exposure and K_ex_, and PS2 following oral exposure were considerably wider than those of calibrated parameters (Table 2), demonstrating that a relative error of less than 5% of C_max_ is insufficient to restrict simulations such that they are consistent with experimental data. Figure 6 shows the numerically derived 95% credible interval (derived using the method described in section on refinement (see Appendix 1) for the concentration response simulations for the first target plasma concentration following inhalation (Figure 6A) and oral (Figure 6B) exposure. Also shown for comparison are 95% credible intervals computed from only the subset of simulations (approximately 10%) where all parameters were within the parameter limits of Table 2. These comparisons demonstrate a much narrower range of curves resulted from the narrower ranges of calibrated parameters, demonstrating the efficiency and value of calibration. However, this comparison also indicates that a second piece of information, perhaps relating to the half-life of MAA following exposure, could be coded as a second criterion for accepting a proposed parameter set and this would achieve a similar result to the formal model calibration.

**Table 3:** Posterior medians and 95% credible ranges for inhalation (ppm) or oral (mmol/kg) exposure and varying model parameters for the six target c_max_ concentrations in venous blood.

**Target C_max_ (mmol/L)**	**Dose**	**BW**	**QPC**	**PS2**	**kex**
**INHALATION DOSE (ppm)**					
0.28	21.59 (13.24, 31.78)	0.249 (0.245, 0.255)	15.53 (7.97, 22.45)	0.808 (0.368, 1.252)	0.0051 (0.0021, 0.0070)
0.55	43.71 (32.46, 60.39)	0.250 (0.246, 0.255)	15.35 (9.16, 21.47)	0.840 (0.345, 1.250)	0.00051 (0.0023, 0.0071)
1.11	81.15 (63.32, 100.95)	0.250 (0.245, 0.255)	16.13 (8.67, 21.89)	0.847 (0.358, 1.281)	0.0053 (0.0021, 0.0073)
2.77	210.13 (165.72, 259.69)	0.249 (0.245, 0.254)	15.47 (8.40, 21.49)	0.843 (0.35, 1.280)	0.0052 (0.0021, 0.0071)
5.55	412.60 (333.12, 521.36)	0.251 (0.246, 0.255)	15.61 (8.31, 22.18)	0.901 (0.347, 1.295)	0.0053 (0.0021, 0.0072)
11.1	793.62 (618.11, 994.52)	0.250 (0.245, 0.255)	15.95 (9.45, 21.31)	0.893 (0.360, 1.311)	0.0050 (0.0022, 0.0070)
**ORAL DOSE (mmol kg**^−1^ **bw)**					
0.28	0.267 (0.143, 0.326)	0.249 (0.245, 0.254)		1.02 (0.446, 1.288)	0.0046 (0.0019, 0.0070)
0.55	0.510 (0.268, 0.639)	0.249 (0.245, 0.254)		0.961 (0.404, 1.305)	0.0045 (0.0019, 0.0072)
1.11	0.920 (0.483, 1.210)	0.249 (0.245, 0.255)		0.941 (0.398, 1.262)	0.0047 (0.0018, 0.0071)
2.77	2.454 (1.264, 3.108)	0.249 (0.245, 0.254)		0.991 (0.409, 1.290)	0.0047 (0.0022, 0.0069)
5.55	4.586 (2.297, 5.860)	0.250 (0.245, 0.255)		0.929 (0.381, 1.208)	0.0047 (0.0020, 0.007)
11.1	9.151 (5.100, 11.759)	0.250 (0.245, 0.254)		0.908 (0.421, 1.191)	0.0047 (0.0022, 0.0071)

### QIVIVE

**Figure 7:**
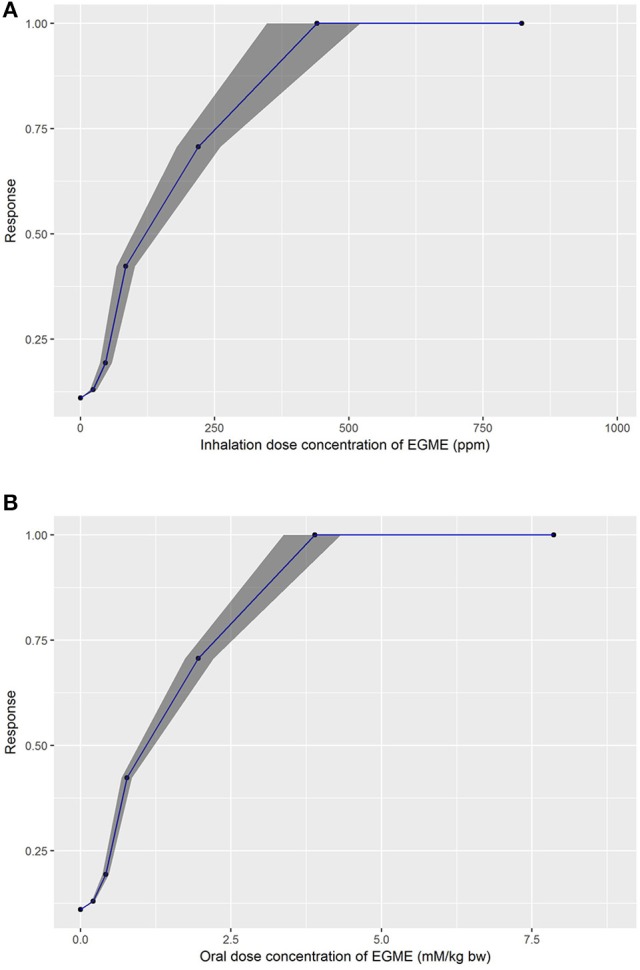
Predicted *in vivo* dose response curves for developmental toxicity of EGME after, **(A)** inhalation (upper panel), and **(B)** oral exposure (lower panel) showing the median, 2.5 and 97.5% percentiles.

The *in vivo* dose-responses estimated from the embryotoxicity, *in vitro* concentration-response data of de Jong et al. (2009) are listed in Table 4. For each target plasma concentration the median and 95% credible interval of external (inhalation or oral) dose were based on the subset of retained simulations that satisfied the relative error criterion and where QPC (only inhalation), K_ex_ and PS2 were within the ranges given in Table 2. As a consequence the intervals for external dose are substantially narrower than the corresponding intervals given in Table 3. The original *in vitro* concentration-response data are also presented for comparison. The dose-response curves for the developmental toxicity of EGME are presented in Figure 7A, inhalation and Figure 7B, oral exposure—these curves correspond to the median (based on the first target plasma concentration) of the generated dose profiles.

**Table 4:** *In vivo* doses estimated by PBPK-based reverse dosimetry.

			**Estimated** ***in vivo*** **doses and exposures**					
	**Number of Non-differentiated embryoid bodies**		**Oral dose EGME (mmol kg**^**−1**^ **bw)**			**Inhalation exposure EGME (ppm)**		
***In vitro* concentation (mM)**	**Mean**	**SD**	**Mean**	**2.5%**	**97.5%**	**Mean**	**2.5%**	**97.5%**
0.00	1.5	0.89	0.00	0.00	0.00	0.00	0.00	0.00
0.28	2.67	2.12	0.21	0.18	0.23	23.67	17.80	30.73
0.55	2.67	2.05	0.42	0.37	0.47	46.69	35.98	58.40
1.11	6.33	3.92	0.77	0.68	0.85	84.12	67.38	101.71
2.77	16.83	3.76	1.96	1.74	2.21	219.72	179.26	260.87
5.55	23.33	1.02	3.89	3.37	4.33	440.41	347.17	521.36
11.10	24.00	0.53	7.86	6.89	8.79	822.10	685.76	986.02

### Benchmark dose analysis

The *in vivo* dose-responses listed in Table 4 were used to derive a BMDL_10_, (lower limit of the 95% confidence interval on the benchmark response equivalent to a 10% effect size), as a point of departure. BMDL_10_ values were derived for the mean, 2.5 and 97.5% percentiles. Comparisons of our predicted BMDL_10_ against BMDL_10_ values derived from *in vivo* studies, for various critical end points, are made in Table 5.

**Table 5:** Comparison of predicted and measured benchmark doses for oral and inhalation exposure.

**Exposure route**	**Days of exposure**	**Dose (mmol kg^−1^ bw)**	**Critical end point**	**Measured BMDL_10_**	**Predicted BMDL**_**10**_				
					**Mean**	**2.5%**	**97.5%**	**Lab 1**	**Lab 2**
Oral	GD7-13	0.3, 0.6, 1.1	Cardiac malformations^a^	0.46	0.32	0.29	0.36	0.12^c^	0.34^c^
			Resorptions^a^	0.52					
			Fetal bw decrease^a^	0.14					
		Dose (ppm)							
Inhalation	GD7-15 (7 h/day)	50, 100, 200	Skeletal malformations^b^	11	34	28	42	21	64
			Visceral malformations^b^	41					
			Resorptions^b^	49					
			Fetal bw decrease^b^	37					

a *Toraason et al. (1985)*.

b *Nelson et al. (1984)*.

c *Louisse et al. (2010)*.

Unlike the study by Louisse et al. (2010) the BMDL_10_ values derived in this study included the laboratory effects as a covariate. Therefore, direct comparisons were not possible. The mean, 2.5 and 97.5% values for both oral and inhalation exposure lie between the lower laboratory 1 and upper laboratory 2 values reported by Louisse et al. (2010) and are similar to the measured BMDL_10_ values. However, the 2.5% value of 0.29 mm/kg bw for oral exposure was still twice that of the lowest measured value of 0.14 mm/kg bw for decrease in fetal bodyweight. Likewise, the 2.5% value of 28 ppm for inhalation exposure was also over 2.5 < the measured value for skeletal malformations.

## Discussion

Various groups have used PBPK modeling-based reverse dosimetry for the translation of *in vitro* to *in vivo* responses using what we termed an *iterative forward dosimetry* approach. This involves fixing all model parameters at baseline values except for some external dose measure (which varies according to route of exposure) and tuning this dose measure such that the predictions from the model are consistent with a target *in vitro* value. Two implicit assumptions are made in such approaches: (1) that the PBPK model is appropriate for the study; (2) that the baseline model parameters assumed in the study are known. Rowland et al. (2017) demonstrated that the level of biological detail contained within a model may affect predictions therefore a model that is broadly consistent with any available experimental data may be insufficient to demonstrate that results are insensitive to model structure. Furthermore, there may be structural deficits in the model, for example the EGME model following repeated low inhalation doses of 10 ppm over-predicted measurements (Figure 3). Structural deficits in the model are sources of *model uncertainty*. The simulations in our own work (Figure 1B) demonstrate that for a given PBPK model structure a range of model parameters might provide a similar quality of fit to data. Even for animal models, where the variability in physiology of the animal is relatively small, the uncertainty in chemical specific parameters predicted using computational tools/models is more substantial (Pearce et al., 2017). Therefore, even for animal models, *parameter value uncertainty* should also be considered when developing a QIVIVE approach. Any methodological approach that fails to account for model and parameter value uncertainty suffers from an important conceptual weakness. Any subsequent inference based upon *in vitro* to *in vivo* extrapolations fails to account for sources of uncertainty and results in over-confident predictions.

The motivation for our approach was to address the shortcomings of simpler approaches and develop a robust methodology that accounted for *structural uncertainty* in the model, including both model fidelity and structural error, *and parameter value uncertainty*. In this work we demonstrate a workflow for the calculation of an *in vivo* point of departure comprising of four steps: (1) GSA to identify the most sensitive model parameters that govern variance of the dose metric; (2) refinement of parameter ranges through model calibration to experimental data; (3) QIVIVE using ABC; (4) calculation of a benchmark dose. The second step in this approach can be eliminated if data for calibration are unavailable; this will result in a greater spread of dose consistent with a target *in vitro* concentration unless further constraints are imposed in the ABC acceptance criteria (alternative constraints are discussed below). The methodology is independent of the *in-vitro* experimental data and could be applied to more advanced experimental approaches (for example utilizing organ-on-a-chip).

We regard accounting for structural error in the PBPK model as an important step in our approach and this is an area where little attention has been invested in work reported in the peer-reviewed literature. The PBPK model is an approximation to reality and the differential equations describing these models inevitably do not encode sufficient detail to replicate the biology of the animal. That is not to discount the utility of such models, but even when predictions are consistent with experimental data, such as in the predictions of plasma concentration of MAA over time following an oral dose to EGME (Figure 4), the prediction is not a perfect fit to data. The scatter of data about the best estimate prediction from the model does not simply reflect measurement error; it also reflects lack of fit which we term model uncertainty. Model uncertainty takes on greater importance when specific outputs from the model (such as peak concentration, area under the curve (AUC), cumulative time above some threshold value or “steady-state” concentration) are extracted from the model for further analysis. When a PBPK model only has to fit a single data point, such as the peak (C_max_) plasma concentration of parent chemical or metabolite, corresponding to a value from *in-vitro* experiments, for fixed values of all other PBPK model parameters it is possible to estimate a unique external dose concentration that results in the target plasma concentration (within the tolerance limits of the optimization routine used). However, to account for model and parameter value uncertainty in our work we accepted external doses that resulted in a relative error for peak plasma concentration of MAA of up to 5%—this was based upon a comparison of model predictions and experimental data (Figures 3, 4).

The range of external dose consistent with each target *in vitro* concentration shows the effect of accounting for sources of uncertainty in QIVIVE (Table 4) with the ranges increasing as the target *in vitro* concentration increases. However, the benchmark dose calculation yielded results similar to those of Louisse et al. (2010) although our approach also produces a credible interval for the benchmark dose lower bound. This broad consistency with Louisse et al. (2010) and the narrow confidence interval for the benchmark dose lower bound probably results from the steep dose relationship found in the experimental data of de Jong et al. (2009) with embryo toxicity, increasing rapidly with dose. In general, for other chemicals and dose metrics such close consistency may not be the case.

Moving onto technical aspects of our methodology we note that in principle parameter value uncertainty could be accounted for using a brute-force Monte Carlo approach, with model parameters sampled and the external dose subsequently optimized such that predictions of plasma concentration were consistent with the target concentration. A distribution of external dose would be estimated for each target plasma dose concentration. However, this approach fails to account for model uncertainty. The ABC algorithm developed in our work accounts for both model and parameter value uncertainty within a computationally efficient approach. In the first phase of our ABC approach we identified a region of parameter space, quantified through a covariance matrix, where solutions were within a specified threshold of the target plasma concentration. A relative error of 7.5% was used in current work. It was a deliberate choice to adopt a more relaxed acceptance criterion in this rejection sampling phase to ensure that the covariance matrix used for proposing moves in the ABC MCMC phase was more diffuse than the covariance matrix of the posterior distribution, thus ensuring the MCMC explored the entire posterior distribution. As noted above, a relative error of 5% was adopted as the acceptance criterion in the ABC MCMC sampling. The acceptance rate for moves, at approximately 60%, was high with a lower acceptance rate in the region 25–40% typically preferred. The high acceptance rate probably resulted from a combination of weakly constrained prior distributions for model parameters and the relatively simple acceptance criterion based solely on C_max_. The relative error of each retained sample was stored as model output and thus allowed for a filtering of accepted samples by differing threshold relative errors—in effect such filtering would reduce the acceptance rate. Whilst the sensitivity of inference to the degree of model error could in principle be explored, this was not pursued in current work.

The acceptance criteria used in our work was based upon C_max_ however alternative dose metrics, such as AUC or steady-state concentration could be readily substituted. Other criteria on the time concentration relationship could also be included to refine the range of acceptable model behaviors—for example a criterion such as half-life within a specified time period following peak exposure or of 95% of MAA cleared within some threshold of a central time after dosing could have been easily included. There is also no need to assume a symmetric error as used in our work; asymmetry in the relative error say 5% above and 2% below the target *in vitro* concentration could easily be coded. Furthermore, the relative error could vary by concentration—this might have been more appropriate for the lowest concentration for the repeat dose inhalation model. Stricter and /or different acceptance criteria would impact upon the range of models behaviors that are considered to be plausible. However, the coding is trivial once the broad behavior of the time concentration relationship has been defined. The sole change would be to the mean and covariance matrix of the model parameters corresponding to the retained samples. Our results (Figure 6) suggest that stricter acceptance criteria could achieve a similar reduction in the range of concentration-time relationships compared with the refinement of sensitive parameters through calibration (step two of our workflow). The mathematics is straight-forward once acceptance criteria are coded, however the process of defining acceptable model behavior is potentially more difficult and could utilize experimental data and expert judgment. The ability to investigate the sensitivity of results to the choice of acceptance criteria is available in our proposed workflow since the samples obtained by MCMC sampling can be filtered by differing acceptance criteria.

The two-phased approach used in our work could be considered “over-engineered” in the sense that the MCMC ABC approach was not strictly necessary for this work since sampling could have been done with sufficient efficiency to make rejection sampling alone feasible, perhaps with some refinement to the drawing of samples (through an iterative approach). The ABC-MCMC approach provides an efficient framework for drawing samples in more challenging examples—where the output metric under study was sensitive to a greater number of parameters—when rejection sampling is too inefficient to be feasible. The proposal distribution specified based on the retained samples from the rejection sampling step could be refined through an adaptive metropolis approach (Rosenthal, 2011). This refinement would be necessary if the acceptance criteria were stringent and the sampling efficiency of rejection sampling was poor.

Finally, we briefly comment on future applications of our approach. An immediate objective is to demonstrate our QIVIVE framework for a human model. This provides a more difficult challenge since there is the need to account for variability in the human physiology and uncertainty (and variability) in chemical specific parameters of the PBPK model. Whilst some aspects of the modeling framework are directly transferable, the ABC approach needs further refinement. Clearly there is a need to limit the candidate parameters such that they correspond to a physically realistic physiology which can be achieved by drawing candidate physiologies from software applications such as PopGen (McNally et al., 2014) or from probabilistic models (McNally and Loizou, 2015), pairing these parameter sets with uncertain chemical specific parameters and dose, and investigating the parameter space of models consistent with the ABC acceptance criteria. A second refinement that is desirable for a human model is the simultaneous estimation of a series of the external doses consistent with the series of *in-vitro* concentrations, conditional on the same physiology all within the same step, thus allowing for the estimation of a concentration response relationship at the level of the individual. In the current approach this matching of samples was achieved after all the MCMC output for each dose concentration had been obtained—a similar approach based upon conditional probability could be implemented to generate dose profiles at each iteration of the chain—the technical details would be developed alongside a suitable example.

A second area of future application is in the estimation of external dose based upon BM data from spot samples. Reverse dosimetry in such applications is typically achieved using the methodology of Tan et al. (2006a) which allows percentiles of the (unknown) exposure distribution to be estimated, but is unable to relate BM data to exposure at the individual level. The methodology developed in this work is directly transferable to this situation and could allow for a more precise analysis of individual risk.

Finally, we briefly comment on implementation of the workflow which requires expertise in global sensitivity analysis and Bayesian statistics (in particular the requirement to hard code a Metropolis-Hastings sampling algorithm). We believe the overall approach can implemented by subject experts based upon the information provided in Appendix 1, although not without significant effort. Some aspects of the workflow (in particular the two-phased GSA and MCMC sampling for parameter calibration) are supported in an easy to use software application, RVis that is based upon the R software platform. The next release due in early 2019 will refine the features of the existing application and add additional capabilities, including optimization and rejection sampling routines. In the medium term our ambition is to support the full workflow demonstrated in this manuscript, at which point it will become more accessible to experts in toxicology who lack a deep understanding of Bayesian statistics.

## Author contributions

KM developed the concept, statistical methodology and drafted and revised the manuscript. AH developed the code and implementation of the workflow and revised the manuscript. GL contributed to the development of the concept, analyzed the data, drafted and revised the manuscript.

### Conflict of interest statement

The authors declare that the research was conducted in the absence of any commercial or financial relationships that could be construed as a potential conflict of interest. The reviewer NS and handling Editor declared their shared affiliation.

## Footnotes

^1^The term PBPK is synonymous with physiologically based kinetic (PBK), physiologically based biokinetic (PBBK), and physiologically based toxicokinetic (PBTK) models.

^2^http://cefic-lri.org/projects/aimt7-rvis-open-access-pbpk-modelling-platform/

^3^Now at KWR (www.kwrwater.nl)

^4^The alternative simulations shown here to motivate the approach correspond to the first 10 retained samples from MCMC sampling using the approach described in “refinement”; in Appendix 1.
