# Supplementary material for: A Computational Workflow for Probabilistic Quantitative in Vitro to in Vivo Extrapolation
Source: Front Pharmacol. 2018 May 18;9:508. doi: 10.3389/fphar.2018.00508 (PMC5968095; doi:10.3389/fphar.2018.00508)
Supplement: Supplementary file 1 [file Data_Sheet_1.DOCX]

**Table S1:** Results from Morris Test OAT sensitivity analysis for the model output of MAA in venous blood following repeat dose inhalation exposure to EGME. Results following inhalation dose concentrations of 10 and 300 ppm at 100 and 124 hours following beginning of the simulation period are given. Parameters taken forward to the second phase of sensitivity analysis are italicised

| Parameter | Label | CONC = 10ppm | | | | CONC = 300ppm | | | |
| --- | --- | --- | --- | --- | --- | --- | --- | --- | --- |
|  |  | µ* | σ | µ* | σ | µ* | σ | µ* | σ |
| *BW* | ***X1*** | 0.017896 | 0.003199 | 0.02236 | 0.003863 | 0.67071 | 0.115867 | 0.537814 | 0.096221 |
| VLC | **X2** | 0.001646 | 0.000237 | 0.000351 | 3.42E-05 | 0.011133 | 0.001184 | 0.052395 | 0.00797 |
| VFC | **X3** | 0.000439 | 9.24E-05 | 0.000535 | 0.000218 | 0.01597 | 0.006543 | 0.012799 | 0.002863 |
| *VSC* | ***X4*** | 0.00196 | 0.000175 | 0.001164 | 8.37E-05 | 0.035581 | 0.002514 | 0.060554 | 0.005443 |
| VRC | **X5** | 0.000239 | 5.74E-05 | 0.000113 | 7.42E-05 | 0.003438 | 0.00222 | 0.007355 | 0.001727 |
| VBC | **X6** | 0.000228 | 6.36E-05 | 0.000374 | 9.32E-05 | 0.011158 | 0.0028 | 0.006597 | 0.001966 |
| QCC | **X7** | 8.78E-05 | 0.000111 | 2.56E-05 | 2.09E-05 | 0.000759 | 0.000625 | 0.002758 | 0.003377 |
| *QPC* | ***X8*** | 0.031436 | 0.001859 | 0.01958 | 0.001657 | 0.588131 | 0.049852 | 0.939902 | 0.055728 |
| QLC | **X9** | 0.000383 | 8.94E-05 | 6.95E-05 | 2.06E-05 | 0.002223 | 0.000481 | 0.011442 | 0.002683 |
| QFC | **X10** | 1.08E-05 | 1.40E-05 | 5.36E-06 | 3.92E-06 | 0.000162 | 0.000118 | 0.000324 | 0.000413 |
| QSC | **X11** | 0.000407 | 8.11E-05 | 5.92E-05 | 8.10E-06 | 0.001785 | 0.000242 | 0.012234 | 0.002435 |
| QRC | **X12** | 4.94E-05 | 6.09E-05 | 1.74E-05 | 1.31E-05 | 0.00052 | 0.000395 | 0.001474 | 0.001846 |
| PB | **X13** | 2.12E-05 | 3.01E-06 | 1.42E-05 | 2.12E-06 | 0.000437 | 6.67E-05 | 0.000649 | 9.25E-05 |
| PL | **X14** | 6.99E-05 | 8.25E-06 | 2.12E-05 | 1.16E-05 | 0.000492 | 5.43E-05 | 0.002158 | 0.000238 |
| PF | **X15** | 0.000109 | 2.41E-05 | 2.60E-05 | 6.02E-06 | 0.000796 | 0.000186 | 0.003344 | 0.000749 |
| PS | **X16** | 0.001534 | 0.000277 | 0.000361 | 5.58E-05 | 0.011093 | 0.001715 | 0.046805 | 0.008686 |
| PR | **X17** | 0.000136 | 1.82E-05 | 3.22E-05 | 6.73E-06 | 0.000987 | 0.000209 | 0.00416 | 0.000542 |
| PL2 | **X18** | 0.000362 | 0.000127 | 0.000407 | 0.000183 | 0.012208 | 0.005493 | 0.01078 | 0.00379 |
| PF2 | **X19** | 0.000161 | 4.54E-05 | 0.000158 | 4.14E-05 | 0.00473 | 0.001241 | 0.004786 | 0.001352 |
| *PS2* | ***X20*** | 0.004687 | 0.001181 | 0.005835 | 0.001248 | 0.174855 | 0.037414 | 0.139236 | 0.035276 |
| *PR2* | ***X21*** | 0.000511 | 8.84E-05 | 0.000611 | 0.000144 | 0.018317 | 0.004308 | 0.015224 | 0.002648 |
| K_M_ | **X22** | 0.001686 | 0.000267 | 0.000384 | 7.72E-05 | 0.011646 | 0.002196 | 0.051707 | 0.008212 |
| V_Max_ | **X23** | 0.001875 | 0.000269 | 0.000423 | 8.27E-05 | 0.013307 | 0.002688 | 0.059556 | 0.008324 |
| *K_ex_* | ***X24*** | 0.018866 | 0.002675 | 0.020041 | 0.002413 | 0.601291 | 0.072425 | 0.565905 | 0.080252 |
| K_up_ | **X25** | 0 | 0 | 0 | 0 | 0 | 0 | 0 | 0 |

**Table S2:** Results from Morris Test OAT sensitivity analysis for the model output of MAA in venous blood following single dose oral exposure to EGME. Results following oral dose concentrations of 0.3 and 3.3 mM at 3 and 19 hours following the beginning of the simulation period are given. Parameters taken forward to the second phase of sensitivity analysis are italicised

| Parameter | Label | ODOSEMMOL = 0.3 mM | | | | ODOSEMMOL = 3.3 mM | | | |
| --- | --- | --- | --- | --- | --- | --- | --- | --- | --- |
|  |  | µ* | σ | µ* | σ | µ* | σ | µ* | σ |
| *BW* | ***X1*** | 0.009462 | 0.001759 | 0.047388 | 0.006831 | 0.090329 | 0.020793 | 0.478635 | 0.081588 |
| *VLC* | ***X2*** | 0.00703 | 0.001965 | 0.001529 | 0.000375 | 0.096484 | 0.062216 | 0.021742 | 0.006507 |
| *VFC* | ***X3*** | 0.009668 | 0.001328 | 0.003017 | 0.000549 | 0.111025 | 0.010908 | 0.040285 | 0.00433 |
| *VSC* | ***X4*** | 0.022352 | 0.004316 | 0.003525 | 0.000946 | 0.255602 | 0.034416 | 0.046389 | 0.015274 |
| VRC | **X5** | 0.001702 | 0.001093 | 0.000312 | 0.000363 | 0.019765 | 0.011219 | 0.00366 | 0.003635 |
| VBC | **X6** | 0.007058 | 0.000964 | 0.002334 | 0.000279 | 0.075346 | 0.01623 | 0.030709 | 0.003481 |
| QCC | **X7** | 0.002837 | 0.00033 | 0.000455 | 9.67E-05 | 0.029723 | 0.00733 | 0.004742 | 0.000735 |
| QPC | **X8** | 5.91E-05 | 4.85E-06 | 4.10E-05 | 4.32E-06 | 0.000744 | 0.00012 | 0.000548 | 8.66E-05 |
| QLC | **X9** | 0.000385 | 0.000232 | 0.00014 | 2.11E-05 | 0.007288 | 0.003147 | 0.001783 | 0.000316 |
| QFC | **X10** | 0.000395 | 9.86E-05 | 6.47E-05 | 9.74E-06 | 0.004503 | 0.001144 | 0.000758 | 0.000129 |
| QSC | **X11** | 0.00125 | 0.000231 | 0.000442 | 8.64E-05 | 0.011575 | 0.002782 | 0.004508 | 0.000686 |
| QRC | **X12** | 0.001345 | 0.000128 | 0.000221 | 3.97E-05 | 0.014327 | 0.002902 | 0.002394 | 0.000431 |
| PB | **X13** | 6.52E-05 | 3.26E-06 | 4.47E-05 | 2.96E-06 | 0.000742 | 0.000107 | 0.000546 | 9.30E-05 |
| PL | **X14** | 0.000194 | 0.000104 | 6.34E-05 | 8.19E-06 | 0.002759 | 0.001571 | 0.000656 | 0.000141 |
| PF | **X15** | 0.000522 | 0.000195 | 8.90E-05 | 2.11E-05 | 0.005237 | 0.002387 | 0.000868 | 0.000141 |
| *PS* | ***X16*** | 0.008665 | 0.001238 | 0.001222 | 0.000121 | 0.102872 | 0.029359 | 0.012054 | 0.002378 |
| PR | **X17** | 0.000515 | 0.000424 | 0.000104 | 3.06E-05 | 0.005369 | 0.00177 | 0.00098 | 0.000236 |
| *PL2* | ***X18*** | 0.006061 | 0.002117 | 0.001508 | 0.000423 | 0.063707 | 0.017499 | 0.020477 | 0.003338 |
| PF2 | **X19** | 0.00255 | 0.000564 | 0.000736 | 0.000205 | 0.024511 | 0.007914 | 0.009011 | 0.002423 |
| *PS2* | ***X20*** | 0.095044 | 0.009728 | 0.025997 | 0.003205 | 1.065197 | 0.123212 | 0.36941 | 0.046469 |
| *PR2* | ***X21*** | 0.009866 | 0.001529 | 0.00238 | 0.000621 | 0.095062 | 0.024777 | 0.032873 | 0.007926 |
| *K_M_* | ***X22*** | 0.008287 | 0.003891 | 0.001638 | 0.000376 | 0.087406 | 0.039406 | 0.014309 | 0.002635 |
| *V_Max_* | ***X23*** | 0.009039 | 0.005127 | 0.00178 | 0.000589 | 0.093643 | 0.033119 | 0.017375 | 0.00234 |
| *K_ex_* | ***X24*** | 0.006707 | 0.001713 | 0.031758 | 0.005897 | 0.061914 | 0.007271 | 0.321167 | 0.033227 |
| K_up_ | **X25** | 0.000522 | 0.00044 | 0.00068 | 3.90E-05 | 0.004836 | 0.004679 | 0.004945 | 0.000983 |

| 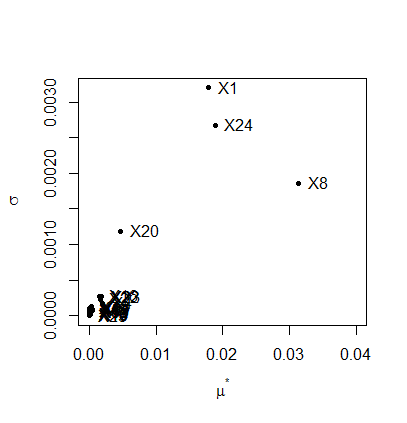 | 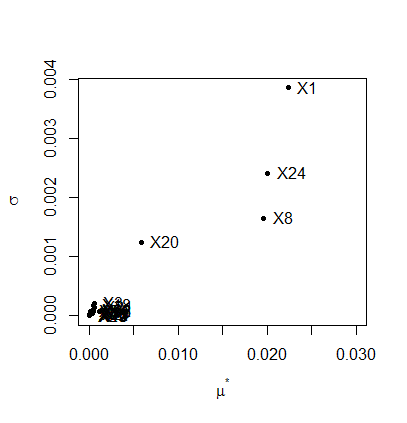 |
| --- | --- |
| 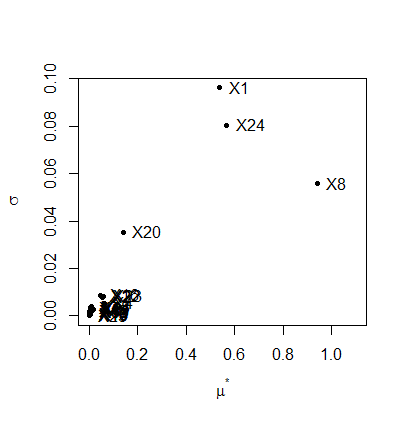 | 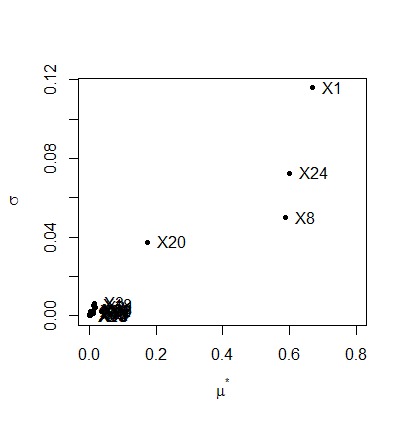 |

**Figure S1:** Comparisons of µ* and σ for the Morris Test OAT sensitivity analysis following repeat dose inhalation exposure

| 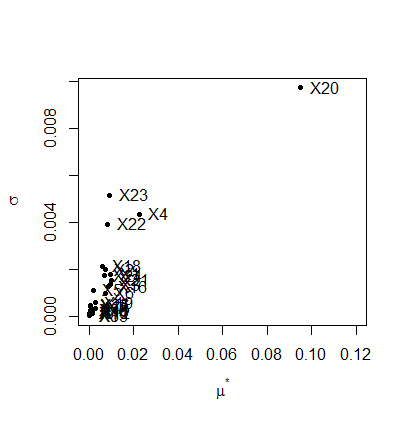 | 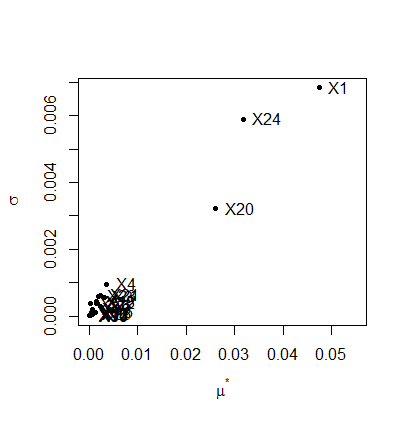 |
| --- | --- |
| 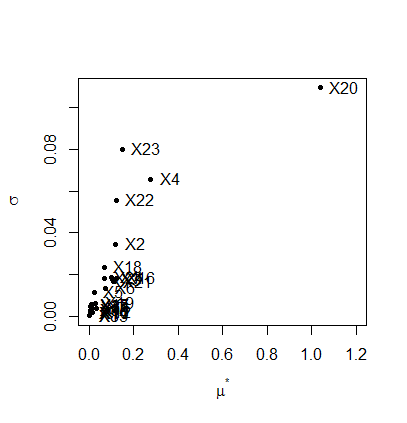 | 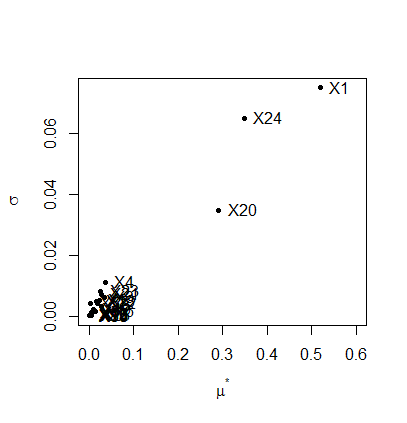 |

**Figure S2:** Comparisons of µ* and σ for the Morris Test OAT sensitivity analysis following single dose oral exposure

|  |
| --- |
|  |

**Figure S3:** Traces plots of main effects (upper panel) and total effects (lower panel) from 0-200 hours for the model of repeat dose inhalation exposure

|  |
| --- |
|  |

**Figure S4:** Traces plots of main effects (upper panel) and total effects (lower panel) from 0-200 hours for the model of single dose oral uptake

| 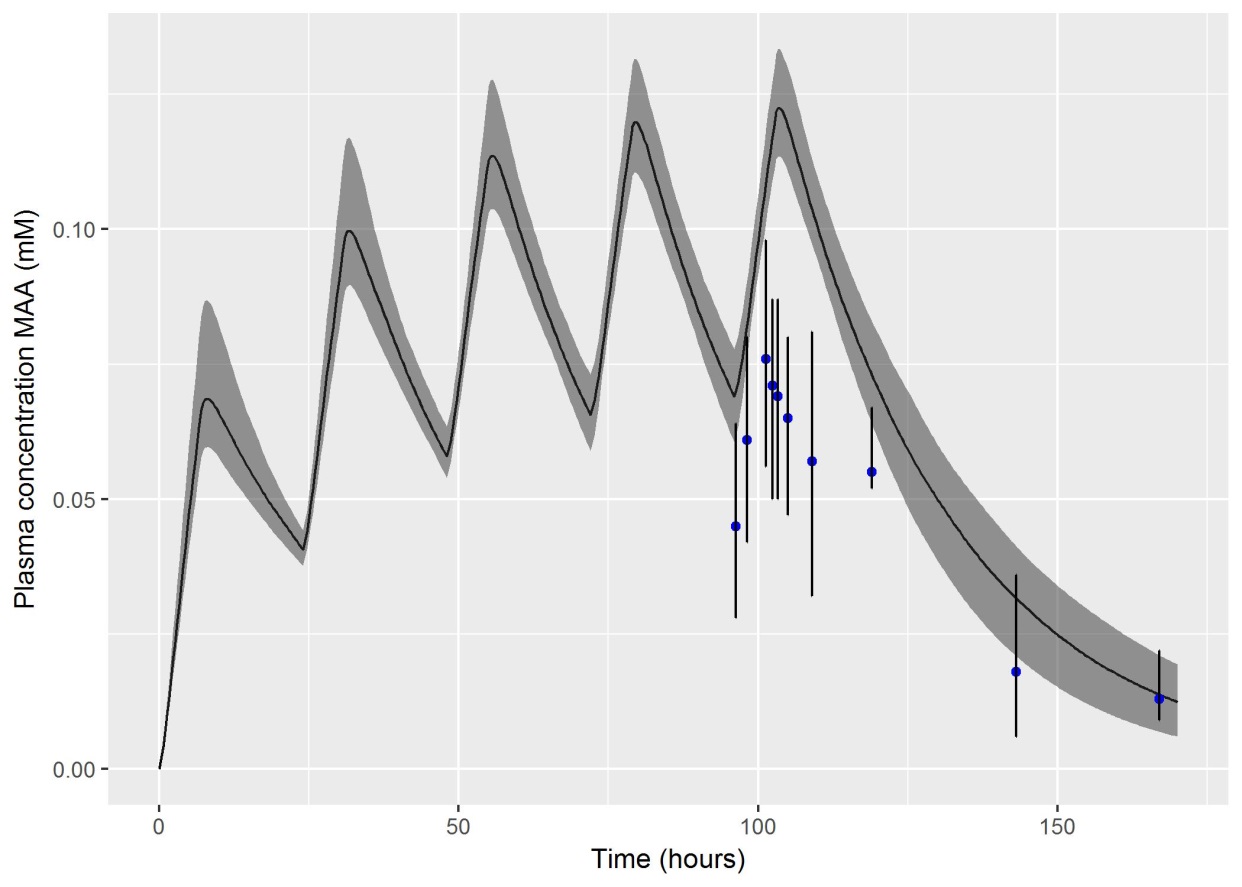 |
| --- |
| 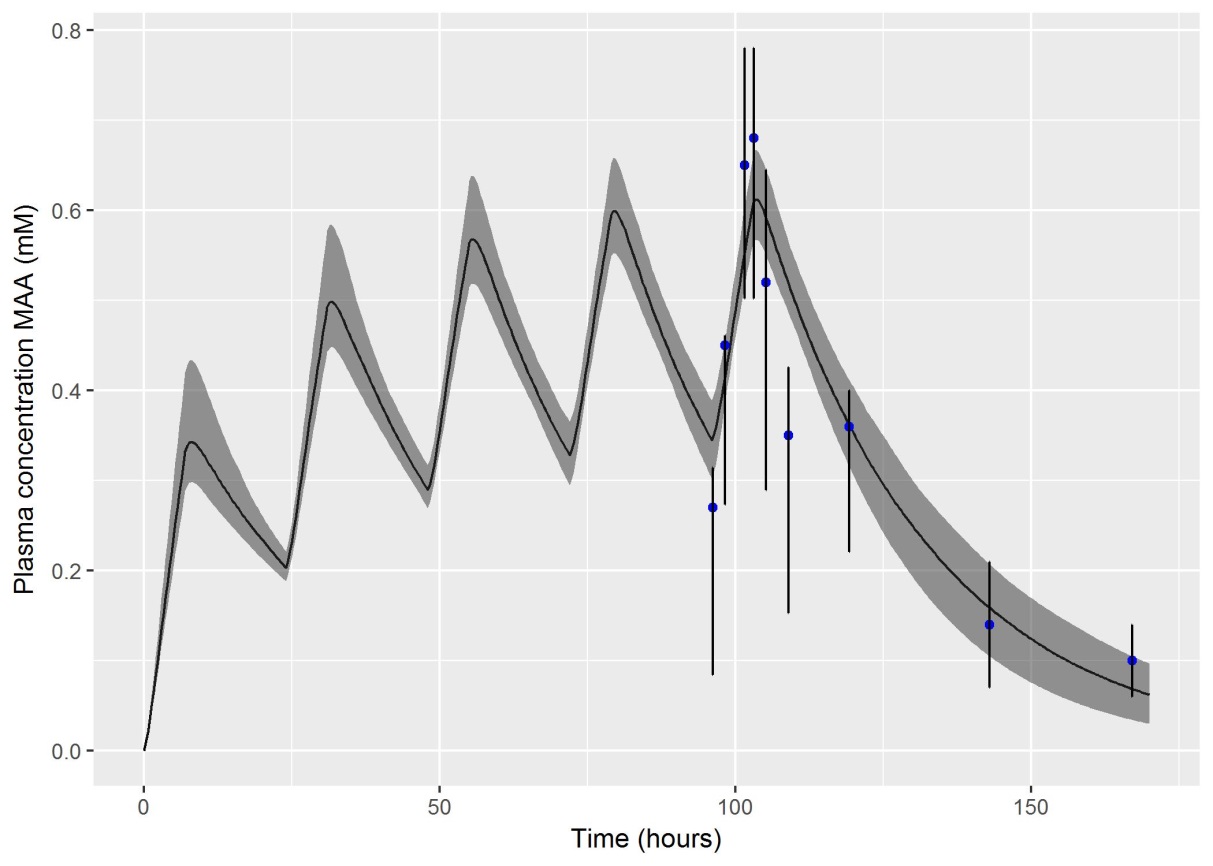 |

**Figure S5:** Posterior mode and a 95% credible interval for the exposure-time concentration of MAA following a 5 day inhalation exposure to 10ppm (upper panel) and 50pmm (lower panel) EGME based upon the alternative Gaussian error model

| 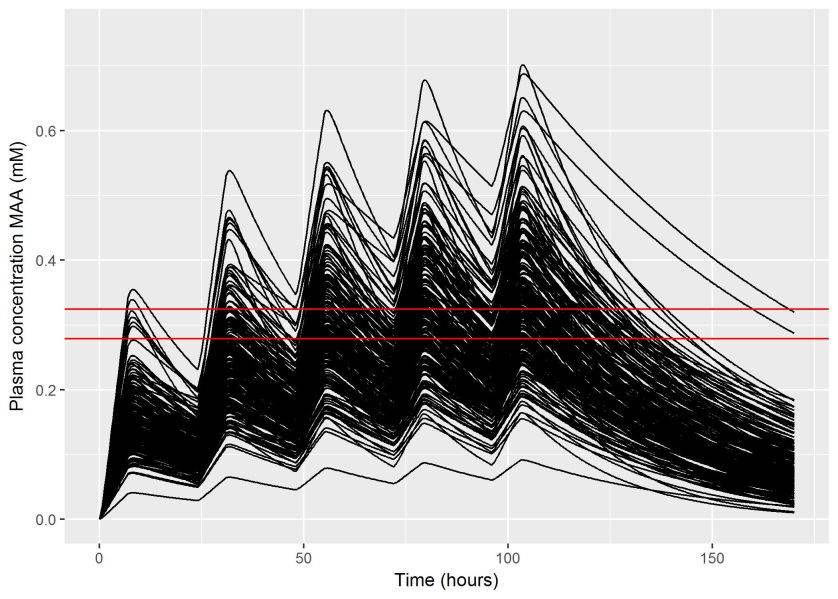 | 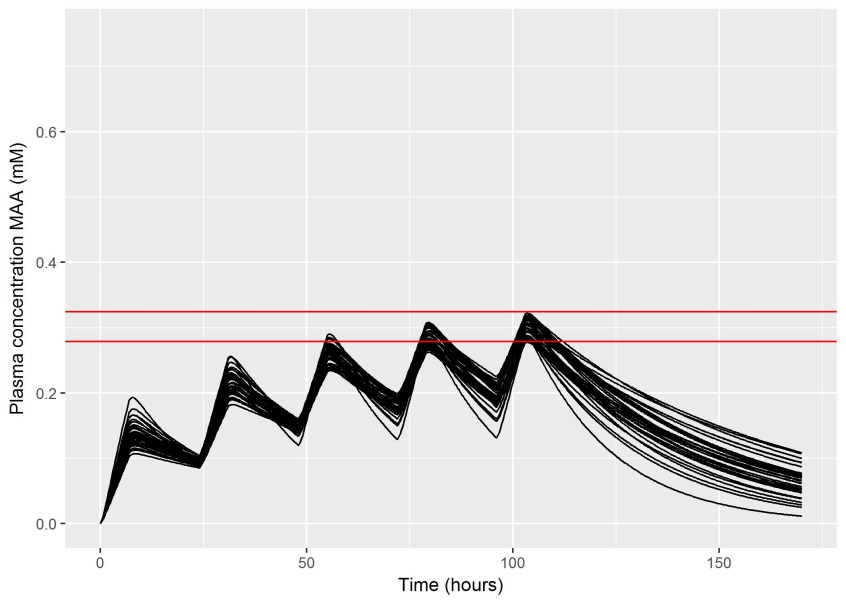 |
| --- | --- |
| 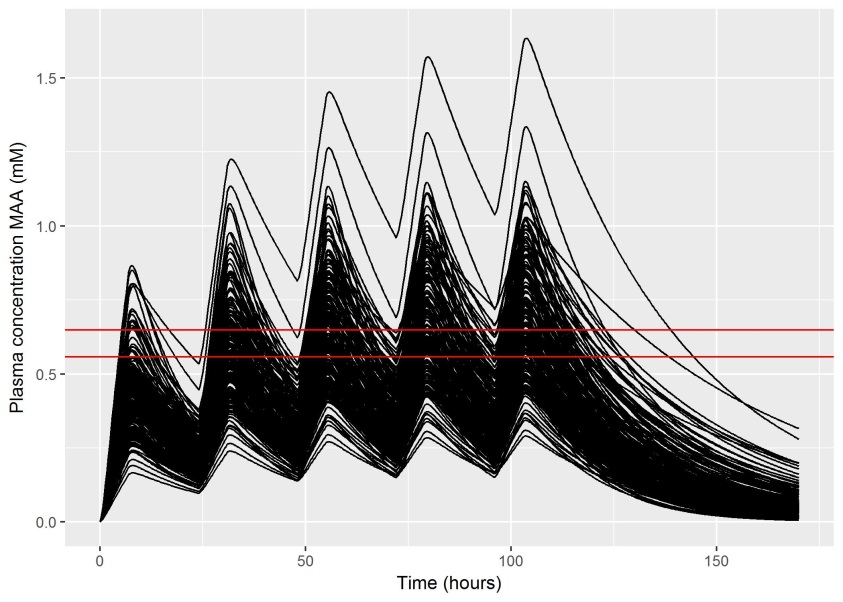 | 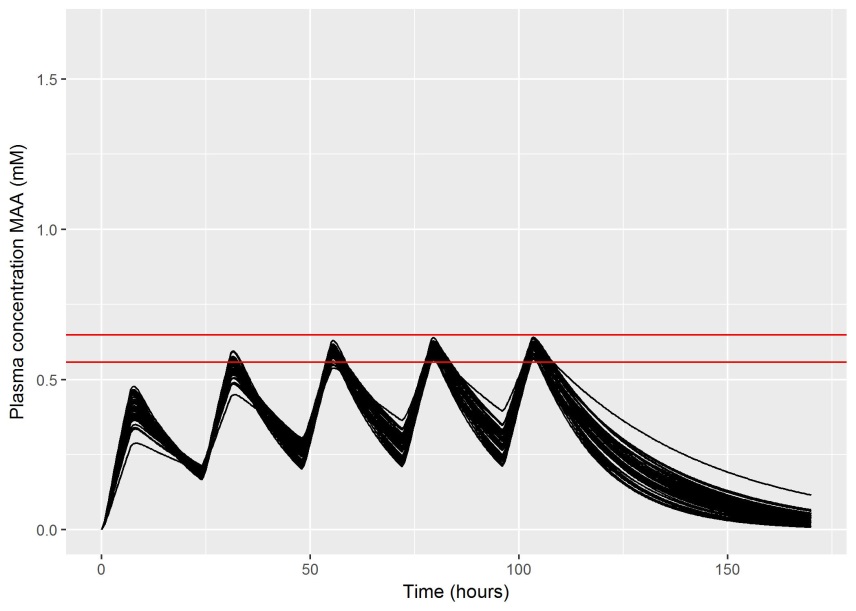 |

**Figure S6**: A) 200 exposure-time concentrations of MAA following repeat dose inhalation exposure for a target Cmax of 0.28 mM; B) retained samples within a relative error of 7.5% (upper right panel); C) 200 exposure-time concentrations of MAA following repeat dose inhalation exposure for a target Cmax of 0.55mM (lower left panel); D) retained samples within a relative error of 7.5% (lower right panel).

| 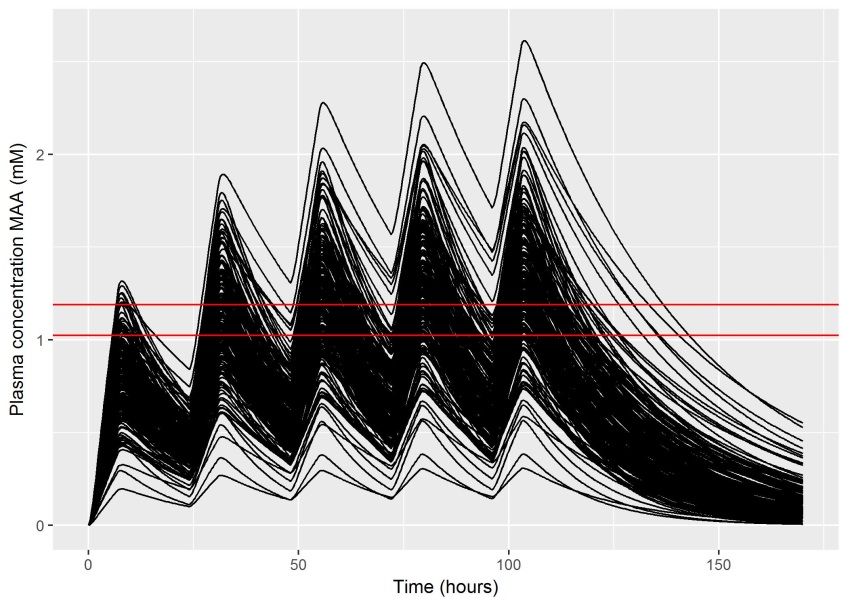 | 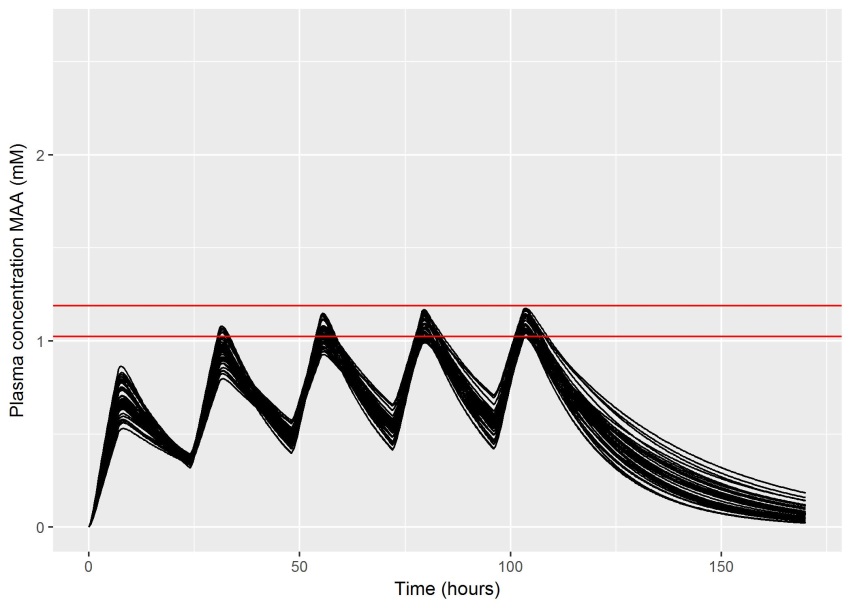 |
| --- | --- |
| 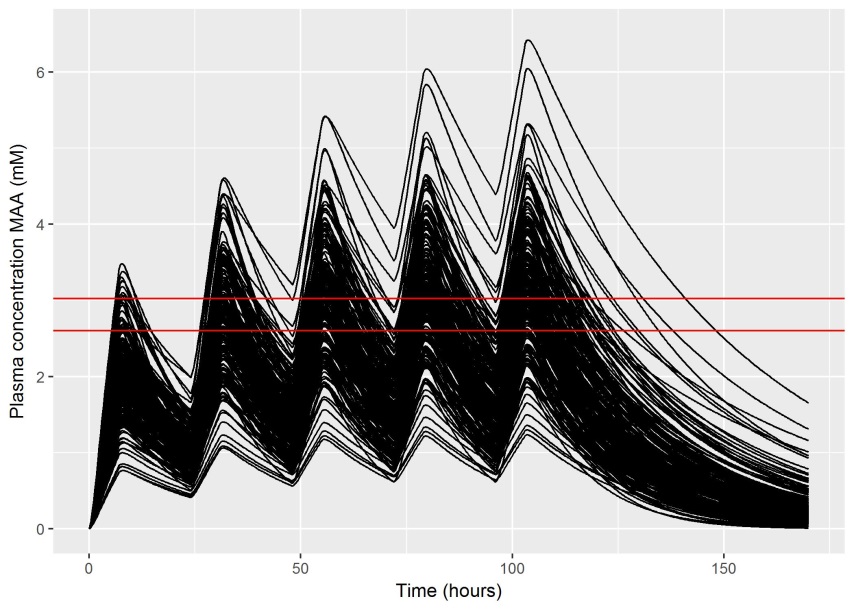 | 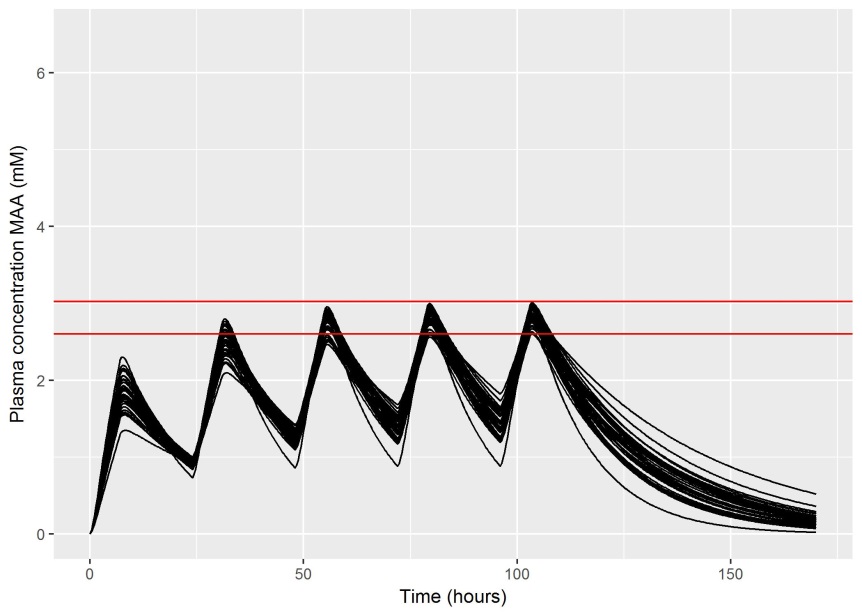 |

**Figure S7**: A) 200 exposure-time concentrations of MAA following repeat dose inhalation exposure for a target Cmax of 1.11 mM; B) retained samples within a relative error of 7.5% (upper right panel); C) 200 exposure-time concentrations of MAA following repeat dose inhalation exposure for a target Cmax of 2.77 mM (lower left panel); D) retained samples within a relative error of 7.5% (lower right panel).

| 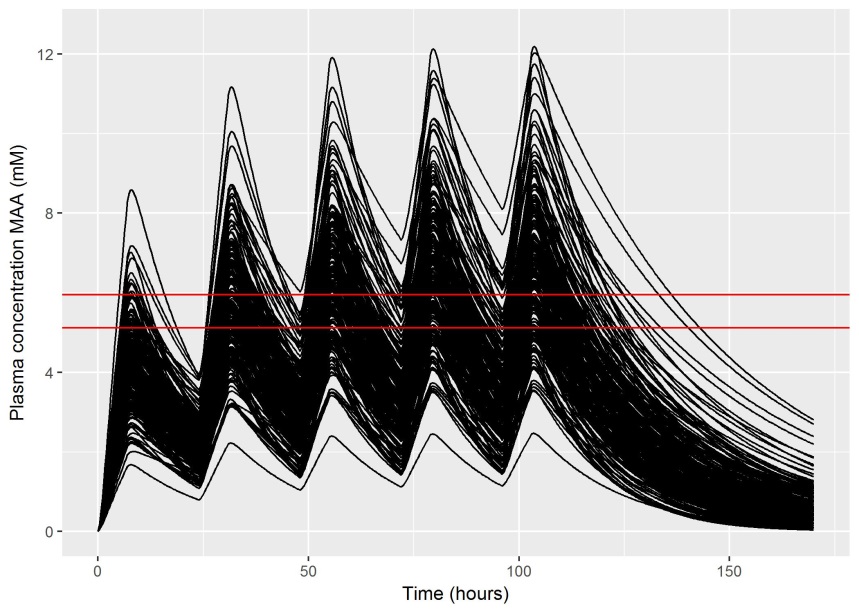 | 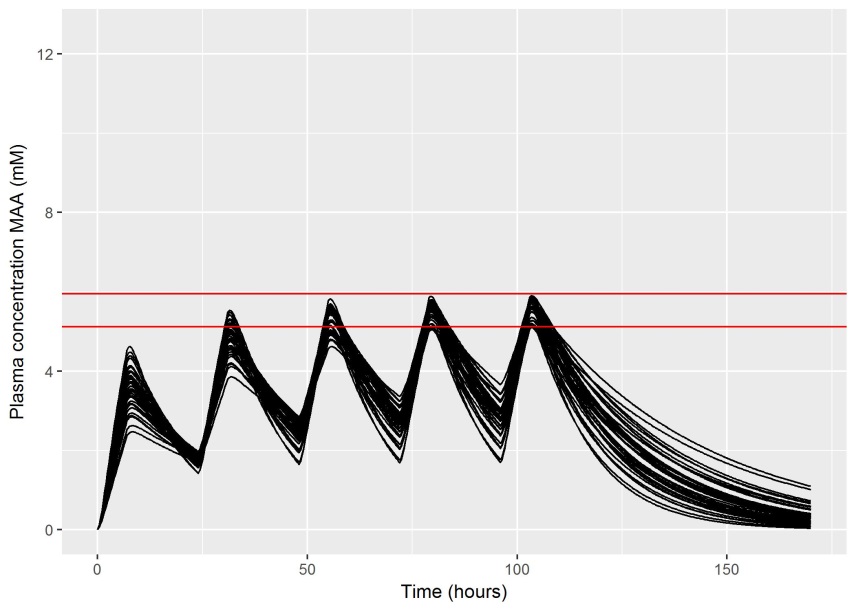 |
| --- | --- |
| 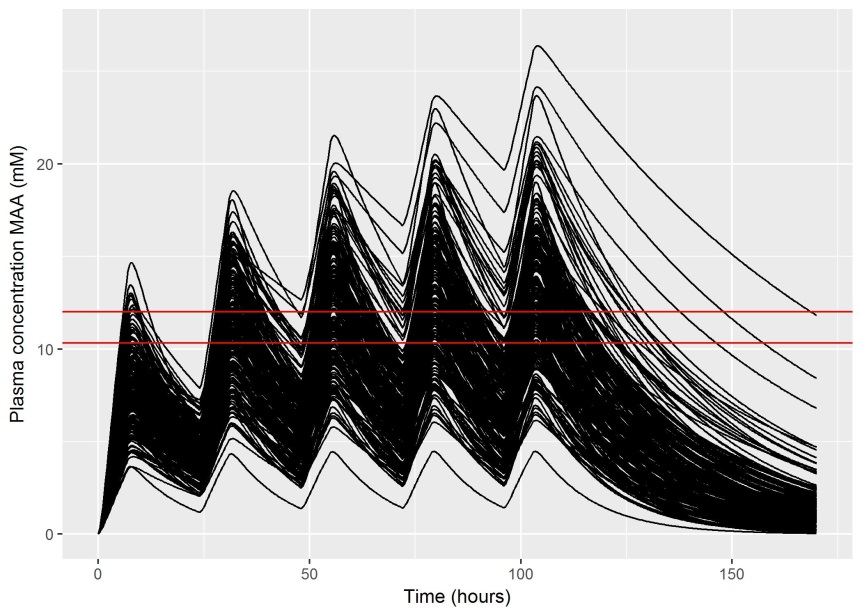 | 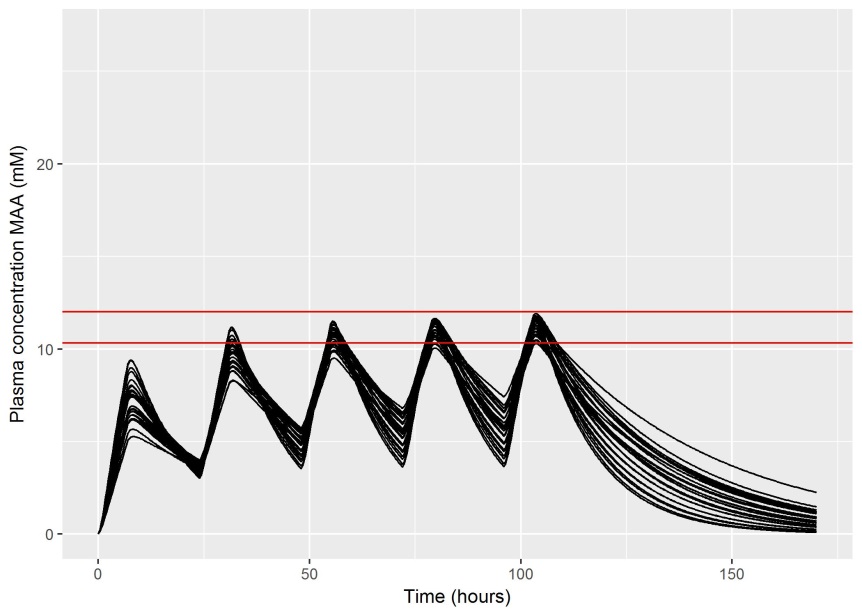 |

**Figure S8**: A) 200 exposure-time concentrations of MAA following repeat dose inhalation exposure for a target Cmax of 5.55 mM; B) retained samples within a relative error of 7.5% (upper right panel); C) 200 exposure-time concentrations of MAA following repeat dose inhalation exposure for a target Cmax of 11.1 mM (lower left panel); D) retained samples within a relative error of 7.5% (lower right panel).

| 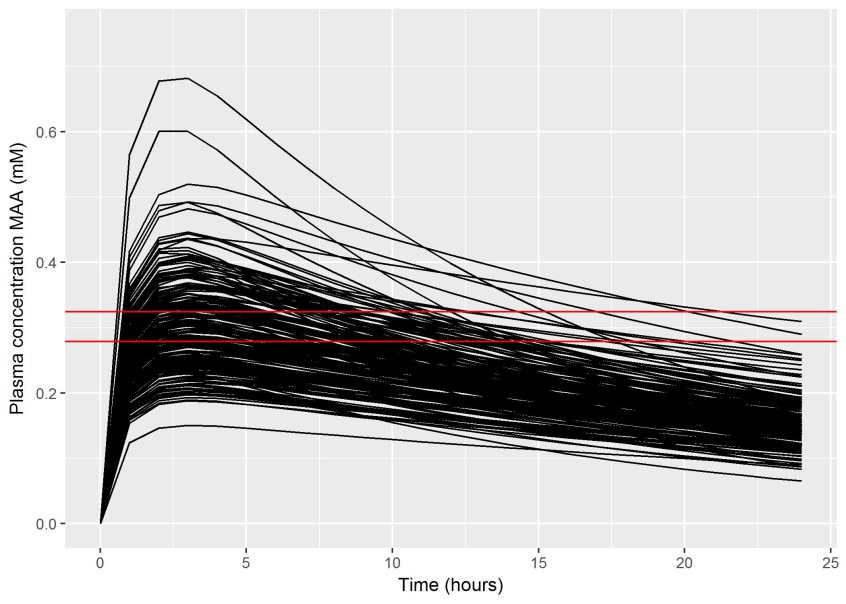 | 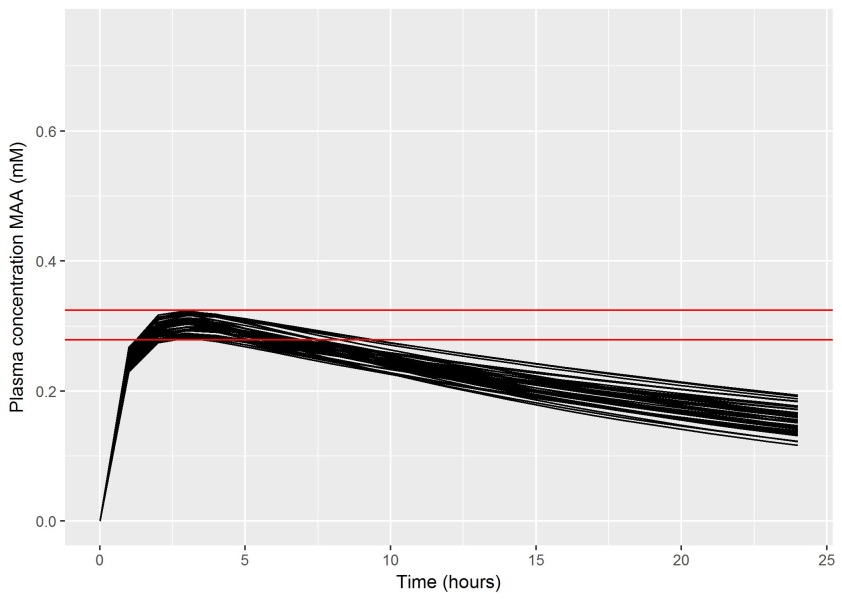 |
| --- | --- |
| 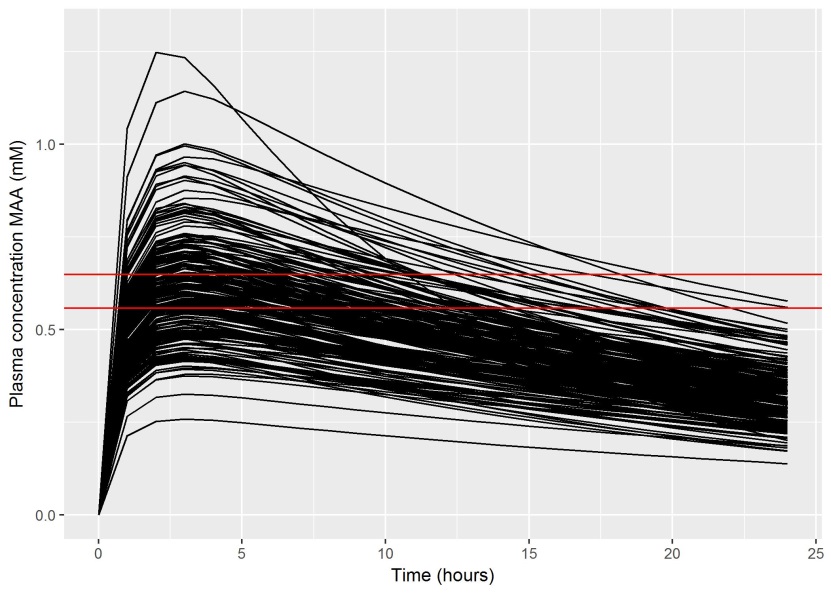 | 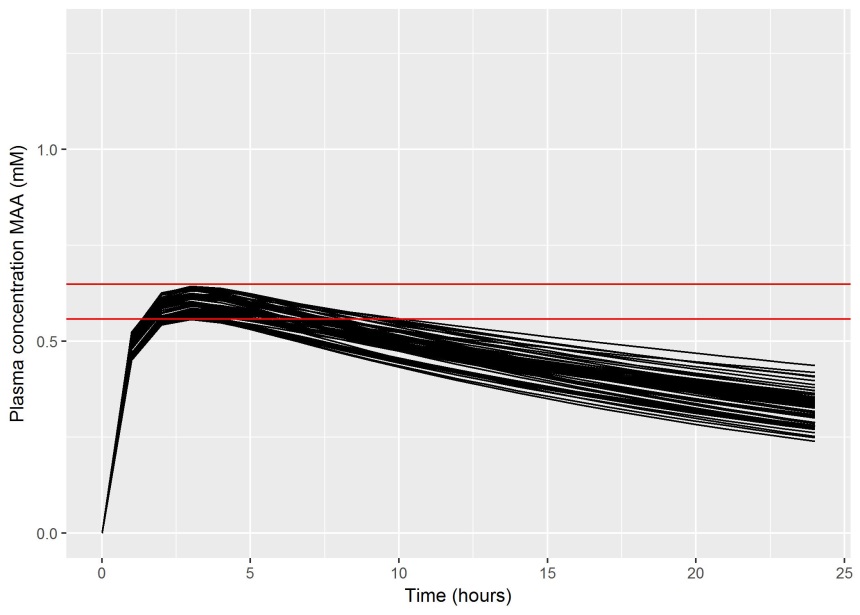 |

**Figure S9**: A) 200 exposure-time concentrations of MAA following single dose oral exposure for a target Cmax of 0.28 mM; B) retained samples within a relative error of 7.5% (upper right panel); C) 200 exposure-time concentrations of MAA following single dose oral exposure for a target Cmax of 0.55mM (lower left panel); D) retained samples within a relative error of 7.5% (lower right panel).

| 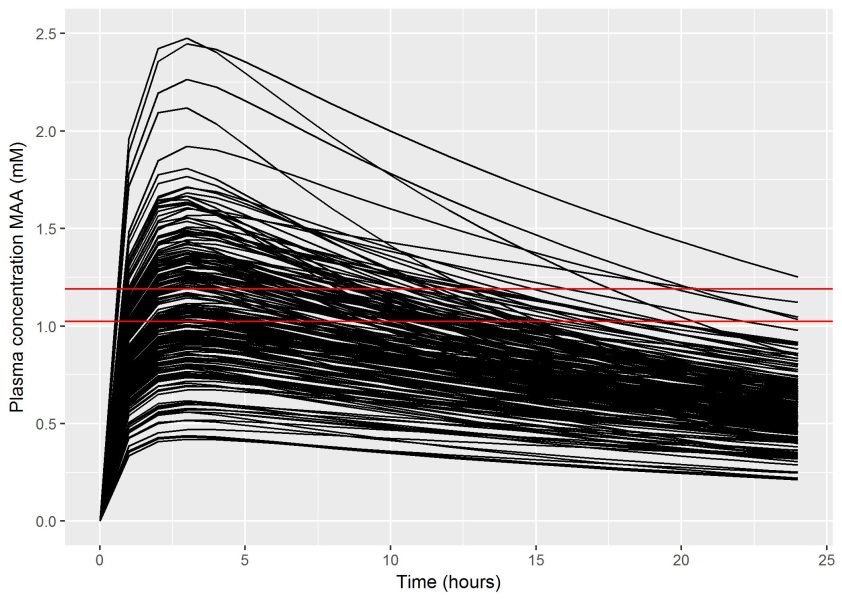 | 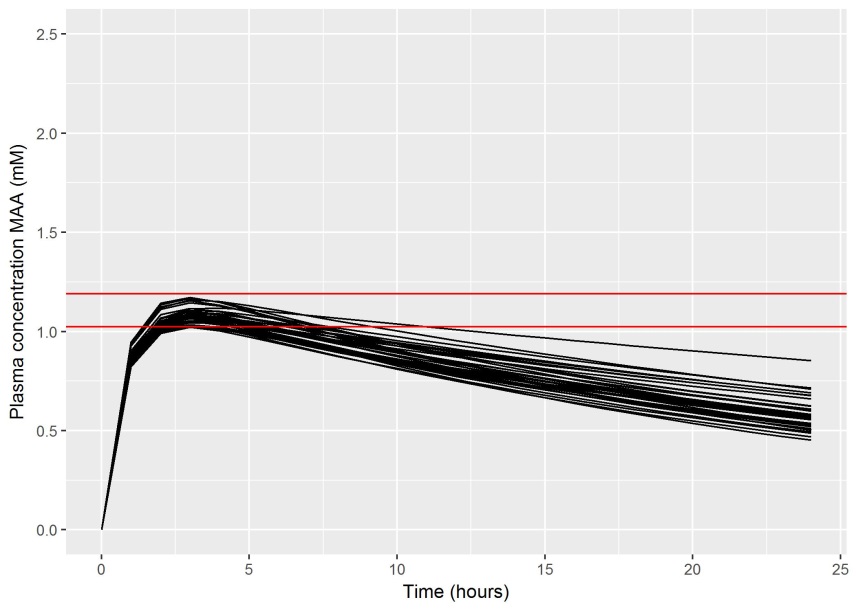 |
| --- | --- |
| 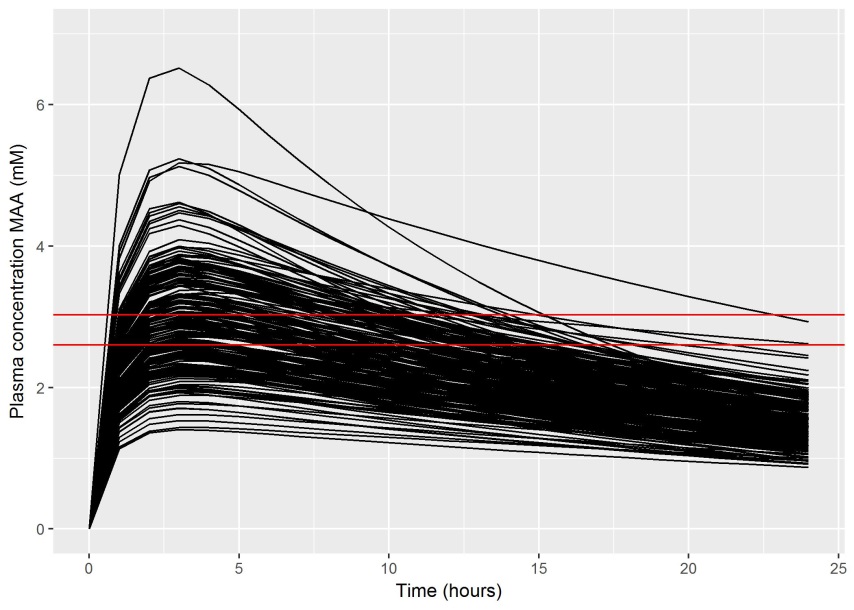 | 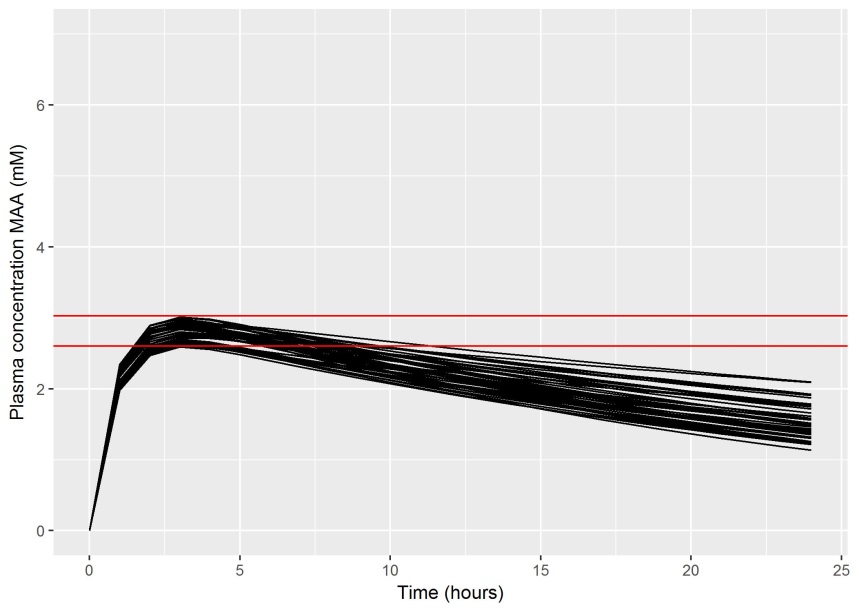 |

**Figure S10**: A) 200 exposure-time concentrations of MAA following single dose oral exposure for a target Cmax of 1.11 mM; B) retained samples within a relative error of 7.5% (upper right panel); C) 200 exposure-time concentrations of MAA following single dose oral exposure for a target Cmax of 2.77mM (lower left panel); D) retained samples within a relative error of 7.5% (lower right panel).

| 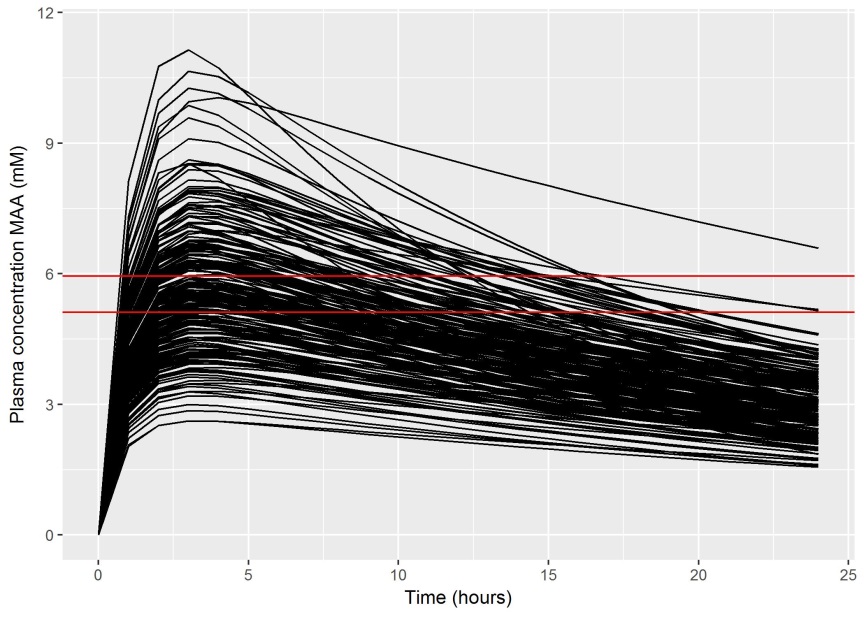 | 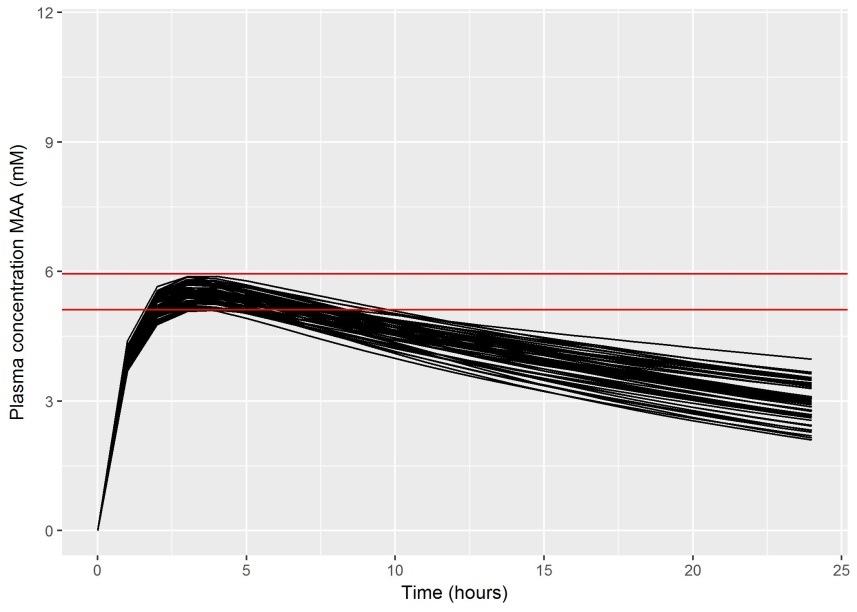 |
| --- | --- |
| 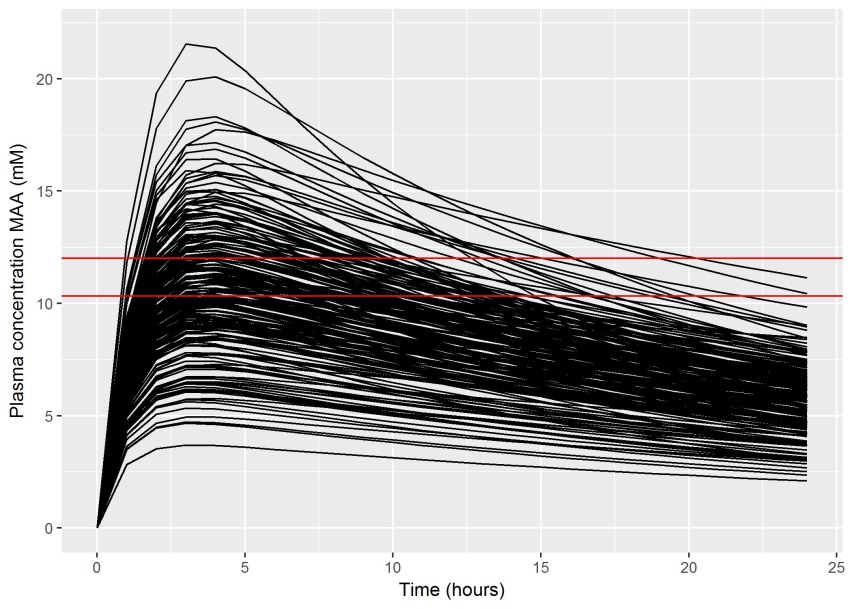 | 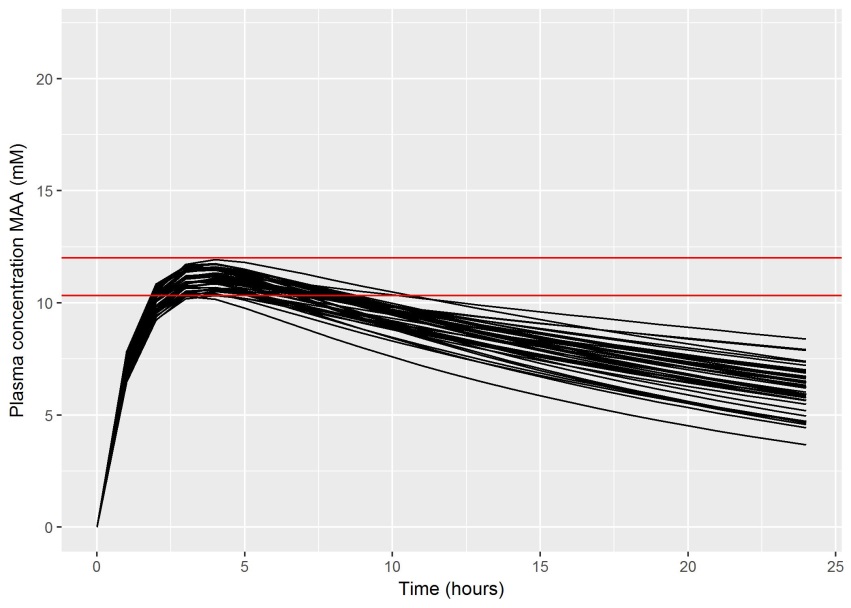 |

**Figure S11**: A) 200 exposure-time concentrations of MAA following single dose oral exposure for a target Cmax of 5.55 mM; B) retained samples within a relative error of 7.5% (upper right panel); C) 200 exposure-time concentrations of MAA following single dose oral exposure for a target Cmax of 11.1 mM (lower left panel); D) retained samples within a relative error of 7.5% (lower right panel).
