# Supplementary material for: A Computational Workflow for Probabilistic Quantitative in Vitro to in Vivo Extrapolation
Source: Front Pharmacol. 2018 May 18;9:508. doi: 10.3389/fphar.2018.00508 (PMC5968095; doi:10.3389/fphar.2018.00508)
Supplement: Supplementary file 2 [file Data_Sheet_2.docx]

# Appendix 1

## GSA

Louisse, et al. (2010) reported the results of a local one at-a-time (OAT) sensitivity analysis for the EGME model. They studied the model output of peak concentration of the metabolite MAA in plasma (venous blood) following a 1 mmol EGME/kg bodyweight oral dose and an eight hour inhalation to EGME at a concentration of 50 ppm. The effect of a 5% increase in each parameter was assessed using normalised sensitivity coefficients. It was noted that the 5% increase was theoretical and did not necessarily correspond to a realistic variation in model parameters.

In this study GSA (GSA) of the EGME model was conducted using the two-phased approach described in McNally, et al. (2011). The computationally efficient Morris test for screening out the parameters with little influence on model output was initially employed. The EGME model was run for 50 hours with results reported at 1 hour intervals following an oral dose of 0.3 and 3.3 mmol EGME/kg bodyweight and following a six hour per day for five days inhalation to EGME at a concentration of 10 and 300 ppm. Lowest and highest oral doses and inhalation exposures were selected to cover the ranges used in the studies investigating EGME toxicity listed in Table 5 of Louisse, et al. (2010).

For both dose concentrations of the oral dose and repeat dose inhalation models the Morris test metrics µ* and σ (see (McNally, et al., 2011) for an interpretation of these parameters) were computed at one hour intervals. An average of µ* and σ over 1 to 4 hours (representing the phase prior to the peak of MAA in venous blood) and 5 to 50 hours respectively (representing the elimination phase of MAA) was computed for the oral exposure model. Averages of µ* and σ computed over the 96 to 102 hour range (representing the uptake of EGME on the fifth day of exposure) and 103 to 200 hours representing the elimination phase of MAA) were computed for the repeat dose inhalation model The aim was to identify parameters with an influence on the maximum concentration of MAA in venous blood (C_max_) at any time point within the simulation period. Those parameters showing no influence on MAA (pairs of µ* and σ close to the (0, 0) point – see Figures S1 and S2 for a graphical demonstration) for either dose concentration or time range were *not* taken forward to the second phase of analysis. The more computationally demanding eFAST technique was used in the second phase with only the sensitive parameters identified in the Morris test varied. Main effects and total effects were computed at each time point and parameter sensitivities were studied over the entire simulation period using Lowry plots (McNally, et al., 2011).

## Parameter ranges

Both the Morris Test and eFAST routines require probability distributions for model parameters to be specified. Uniform distributions, parameterised by a minimum and maximum, were assumed for all parameters. Conservative yet credible ranges for model parameters were chosen. Given that laboratory rats are bred to minimise variability the minimum and maximum ranges for the anatomical and physiological parameters of the rat were set to ±15% of the mean values*.* For simplicity, the partition coefficients, metabolic rates and other rate constants were also given ranges of ±15% of the mean values. For the parameters with little influence on the model output the generous ranges assumed in sensitivity analysis were of no consequence – in subsequent phases of modelling these parameters were held at baseline (central estimate) values. The ranges for sensitive parameters were refined in the second phase of modelling described in the section on refinement.

## Refinement

The second step of our approach is the refinement of parameter ranges for the sensitive parameters identified using GSA techniques. Even though the ranges for sensitive model parameters are narrow these still index an infinite number of parameter sets (McNally, et al., 2012). Calibration describes the process of refining these ranges and therefore narrowing the range of parameter space that is considered to be plausible. This was achieved through a statistical calibration model which provides a formal statistical framework for a comparison of (parameter dependent) PBPK model predictions and measurements available from the experimental data of Hays, et al. (2000)) and Gargas, et al. (2000).

A log-normal calibration model (1) was specified for this comparison, where $\mu_{i}$denotes the PBPK model prediction at times $i=1..T$ corresponding to model parameters$\theta$, $y_{i}$ denotes the corresponding experimental data at times $i=1..T$ and $\sigma$ is a statistical parameter quantifying the disagreement between predictions and measurements.

The raw experimental data of Hays, et al. (2000)), representing a measurement on each of three or four rats which were euthanized at the point of measurement, and Gargas, et al. (2000), representing four animals which were euthanized at the point of measurement, were unavailable to us. However, means and error bounds representing one standard deviation were digitised based upon Figures 2A and 2B of (Louisse, et al., 2010). Calibration was to the dataset of means in each case; however the bounds representing one standard deviation are shown in results.

$f\left( y | \theta,\sigma\right)=\prod_{i=1}^{T} \frac{1}{\sqrt{2\pi\sigma^{2}}}exp\left[ -0.5\left( \frac{log(y_{i})-log(\mu_{i})}{\sigma} \right)^{2} \right]$ (1)

The Bayesian approach described in McNally, et al. (2012) was applied. Uniform prior distributions for sensitive parameters in the PBPK model were assumed. For bodyweight the lower and upper limits were adjusted to 0.245 and 0.255kg respectively to match the range of masses of the experimental rodents. The physicochemical-parameters were assigned priors of half and two times the central estimates given in Table 1 (reflecting greater uncertainty) whereas the limits of **Error! Reference source not found.** were applied for other retained parameters. A half normal prior distribution truncated at zero and with standard deviation of 10 (weakly informative) was assumed for$\sigma$. Inference for model parameters $\theta$ and statistical parameter $\sigma$ was made using an MCMC algorithm (bespoke Metropolis-Hastings algorithm coded in R). The MCMC algorithm was burnt in for 1000 iterations and sampling was conducted for a further 10000 iterations for each model. Every 20^th^ sample was retained for analysis. Checks were made to ensure convergence of the chains and that the auto-correlation was reasonable.

## QIVIVE approach

### Theoretical basis of the approach

MCMC is a well-established approach for drawing samples from the marginal posterior distribution of external dose (represented as a parameter within the PBPK model) from biological monitoring data or some alternative end-point (Lyons, et al., 2008; McNally, et al., 2012). A Metropolis-Hastings algorithm requires values for the external dose and other unknown parameters to be proposed, and accepted as a sample from the posterior distribution if the proposed value is consistent with data. A more rigorous mathematical description of the approach is given in Brooks *et al.* (2011). A requirement of this approach is a statistical error model specified through a likelihood function linking observations to model predictions. A log-normal model (equation 1) is usually assumed for the error structure (McNally, et al., 2012).

For the QIVIVE problem considered in this work, for fixed values of all parameters in the model with the exception of oral (or inhalation) dose, a unique oral (or inhalation) exposure that results in the target C_max_ can be estimated^[[1]](#footnote-1)^ therefore error and hence a likelihood are undefined. As there is no statistical mechanism for discriminating between different parameter sets that provide a perfect fit to C_max_, a conventional MCMC based approach cannot be pursued.

The approach we adopt therefore follows a Likelihood free or ABC MCMC approach (Hartig *et al.*, 2011; Sisson and Fan, 2011) which is a technique that has been developed for problems where the likelihood function is undefined or is difficult or costly to sample from, although summary statistics can be calculated. Within this framework a proposed parameter set is accepted as a draw from the posterior distribution if the summary statistics calculated based upon the proposed parameter set lie within some specified threshold of the summary statistics calculated from data. Within our approach we accept a parameter set if C_max_ calculated based upon the proposed parameter set lies within a specified threshold of the target C_max_. This alternative acceptance criterion replaces the conventional acceptance criterion in a Metropolis Hastings algorithm. Such an approach is theoretically justified by model uncertainty (**Error! Reference source not found.**B).

## Application of ABC MCMC approach

The approach outlined below was repeated for each of the six target in-vitro concentrations

1. An initial 200 evaluations of the PBPK model were made. The parameter sets for these models were sampled from uniform distributions with the same limits as assumed in the section on Refinement. An oral or inhalation dose was paired with each draw: dose was sampled from a uniform distribution with conservative lower and upper bounds (which varied by target concentration) based upon exploratory simulations*.* The refined ranges given in **Error! Reference source not found.** (after calibration) would in general be more appropriate, however an objective of this work was to generate a wide range of concentration response profiles and to assess whether further constraints on concentration-response profiles, in addition to that imposed on C_max_, might offer a viable approach for reducing uncertainty in external dose in cases where data for a formal calibration were unavailable.
2. The C_max_ corresponding to each run was extracted from model output and a comparison of the calculated and target C_max_ was made. The subset of parameter sets where the relative error was within 7.5% (i.e. those satisfying condition (2) were retained and the mean $\boldsymbol{\mu}$ and covariance matrix ∑ were calculated based on this retained subset. This initial analysis preceding the ABC MCMC stage provided information on the subset of parameter space containing ‘good’ solutions.

${{(C}_{max,observed}-C_{max,target})}/{C_{max,observed}}$ < 0.075 (2)

1. Initial values, $\boldsymbol{X}_{0}$ for the Markov Chain were sampled from a multivariate normal distribution with mean and covariance estimated from retained samples (step 2).
2. For subsequent iterations of the ABC MCMC algorithm a proposed parameter set, $\boldsymbol{X}^{'},$ from the target posterior distribution was proposed from a multivariate normal distribution

$\boldsymbol{X}^{'} \sim MVN(\boldsymbol{X}_{n-1},\sum$),

where the proposed move is centred on the current state of the chain (specified through the mean) and with covariance matrix ∑.

1. The C_max_ corresponding to proposed move $\boldsymbol{X}^{'}$ was calculated and the move was accepted as $\boldsymbol{X}_{n}$so long as the relative error satisfied the acceptance criterion and the proposed move was within the bounds used for generating the samples for rejection sampling (step 1). If these conditions were not met then the current state was retained $\boldsymbol{X}_{n}=\boldsymbol{X}_{n-1}.$ The algorithm was run for 5000 iterations.

A relative error of 5% was specified for acceptance – stronger criteria than (2); this was chosen since models that were consistent with experimental data over the time course of experimental data (Figure 1B) under-predicted the peak dose observed in the data by approximately 5%. However, the option of investigating a stricter quality of fit through filtering the samples when post-processing output was available. A fuller discussion of the technical aspects of the ABC MCMC approach is given in the discussion.

## Generating dose profiles

The ABC-MCMC approach described above was independently implemented for each of the six target in-vitro dose concentrations – the MCMC output was therefore independent for each concentration. However, for a given rat physiology (fixed values of all parameters), if the estimated external dose corresponding to the first target in-vitro concentration is large (relative to the posterior distribution of external doses for the concentration), the external doses corresponding to the remaining five target in-vitro concentrations are also likely to be large (relative to the posterior distributions of external doses for the respective concentrations). A rank correlation structure was estimated and the MCMC output was ordered such that the samples had the target correlation structure with each set of six samples of external dose corresponding to a credible dose profile that could be compared with in-vitro data.

The correlation structure was estimated using the retained samples from rejection sampling – the subset of samples for each dose concentration that satisfied the condition (2). The retained samples (for each concentration) were approximated by a multivariate normal distribution with mean and covariance estimated from the data. The conditional distribution of external dose given all the other uncertain parameters was derived (standard theory). The joint probability distribution of the physiological parameters was also multivariate-Gaussian, and common for all six target in-vitro concentrations; 500 samples of the physiological parameters were simulated and the external dose was simulated for all six target in-vitro concentrations resulting in 500 samples of external dose for each in-vitro concentration. Pairwise spearman rank correlations were calculated using the simulated concentrations for each combination of external dose concentrations resulting in a six-by-six matrix of rank correlations. The MCMC samples corresponding to the six in-vitro concentrations were re-ordered such that the samples had the target rank correlation using the method of Iman and Conover (1982).

# Appendix 2

## Script files to implement conversion of in vitro concentration to in vivo dose response

After conversion of the existing PBPK model from CSL to R syntax and modification to implement logical constraints on mass balance and blood flow to the tissues, the approach consisted of the following steps: (1) GSA of the model to identify the most sensitive parameters governing variance in venous blood MAA concentrations, (2) evaluation of the model using time-dependent changes in blood MAA concentrations, (3) estimation of in vivo dose-response curves from in vitro concentration-response data using ABC and MCMC sampling with ranges around the most sensitive parameters only, and (4) calculation of a benchmark dose using the posterior estimates of the in vivo dose-response curves.

The approach was expedited using five bespoke script files used to configure and run the PBPK model, comprising optimization, rejection sampling, ABC-MCMC sampling, calculation of results and plotting charts.

### Optimization

The purpose of the optimization step is to estimate the exposure (oral dose or inhalation concentration) using the baseline (point estimate) values of the parameters in the PBPK model. Before carrying out the optimization, this step attempts to correctly configure the simulation and integration based on the mode of exposure. This file implements the method by which a (maximum) value of the target output is chosen in a prescribed time range; this method is utilized both in the optimization and also in the MCMC implementation. The step makes use of R’s built in **optimize** application programming interface (API) and the result is a list of exposure values, one for each *in vitro* concentration.

### Rejection

The purpose of the rejection step is to draw random parameter values based on the prior (the point values of the parameters), to assess the fit of those values by running the model against them followed by an ABC rejection algorithm, and to compute summary statistics and a covariance matrix on the accepted values.

Random values are drawn from a normal distribution, with the standard deviation estimated based on either the constraint range, or on an assumed interquartile range obtained from the parameter’s point value. For each set of parameters, a fit value is computed from the output maximum. Using a pre-defined accept rate, a proportion of the fitting parameter sets are taken forward.

### ABC-MCMC analysis

A script file executes an ABC-MCMC analysis with priors based on the summary statistics acquired using the rejection method.

The implementation is, in principle, similar to that of a basic Metropolis-Hastings MCMC, but with the acceptance and likelihood functions replaced by an ABC acceptance. Starting values for each chain are chosen randomly from the constraint ranges set up in Step 1 (Optimization). The proposal for each Monte Carlo (MC) iteration was generated using R’s multivariate random normal API. Using a gradual back-off strategy, the MCMC loop adapts the target fit based on the accept rate; in other words, at increasingly wider intervals, the overall accept rate for the chain is assessed and the fit adjusted to keep the rate on target. Multiple chains are run concurrently using the facilities in R’s parallel package.

### Results

This script is used to analyse the chains generated by ABC-MCMC, and to compute the overall accept rates.

Chain data is processed using the R CODA package. Various diagnostic plots are produced to allow for assessment of the efficacy of MCMC sampling.

### Graph Plotting

This script is used to combine the chain data, to select parameter sets that meet the fit criteria, to compute useful statistics on the combined parameter values, to plot the distributions generated by the ABC-MCMC, and to plot the dose-response curves.

1. Estimated in the sense that optimization routines find a solution within a user specified tolerance [↑](#footnote-ref-1)
